# B7H3 targeting gold nanocage pH-sensitive conjugates for precise and synergistic chemo-photothermal therapy against NSCLC

**DOI:** 10.1186/s12951-023-02078-9

**Published:** 2023-10-17

**Authors:** Bing Chen, Kaifan Zheng, Shubin Fang, Kangping Huang, Chengchao Chu, Junyang Zhuang, Jin Lin, Shaoguang Li, Hong Yao, Ailin Liu, Gang Liu, Jizhen Lin, Xinhua Lin

**Affiliations:** 1 Key Laboratory of Nanomedical Technology (Education Department of Fujian Province), School of Pharmacy, Fujian Medical University, Fuzhou, 350122 China; 2 Department of Pharmaceutical Analysis, School of Pharmacy, Fujian Medical University, Fuzhou, 350122 China; 3 The Cancer Center, Union Hospital, Fujian Medical University, Fuzhou, 350122 China; 4 State Key Laboratory of Molecular Vaccinology and Molecular Diagnostics & Center for Molecular Imaging and Translational Medicine, School of Public Health, Xiamen University, Xiamen, 361102 China; 5 The Department of Otolaryngology, Head and Neck Surgery, University of Minnesota Medical School, Minneapolis, 55404 USA

**Keywords:** B7H3/CD276, Gold nanocage, Doxorubicin conjugates, Chemo-photothermal therapy, NSCLC

## Abstract

**Background:**

The combination of drug delivery with immune checkpoint targeting has been extensively studied in cancer therapy. However, the clinical benefit for patients from this strategy is still limited. B7 homolog 3 protein (B7-H3), also known as CD276 (B7-H3/CD276), is a promising therapeutic target for anti-cancer treatment. It is widely overexpressed on the surface of malignant cells and tumor vasculature, and its overexpression is associated with poor prognosis. Herein, we report B7H3 targeting doxorubicin (Dox)-conjugated gold nanocages (B7H3/Dox@GNCs) with pH-responsive drug release as a selective, precise, and synergistic chemotherapy-photothermal therapy agent against non-small-cell lung cancer (NSCLC).

**Results:**

In vitro, B7H3/Dox@GNCs exhibited a responsive release of Dox in the tumor acidic microenvironment. We also demonstrated enhanced intracellular uptake, induced cell cycle arrest, and increased apoptosis in B7H3 overexpressing NSCLC cells. In xenograft tumor models, B7H3/Dox@GNCs exhibited tumor tissue targeting and sustained drug release in response to the acidic environment. Wherein they synchronously destroyed B7H3 positive tumor cells, tumor-associated vasculature, and stromal fibroblasts.

**Conclusion:**

This study presents a dual-compartment targeted B7H3 multifunctional gold conjugate system that can precisely control Dox exposure in a spatio-temporal manner without evident toxicity and suggests a general strategy for synergistic therapy against NSCLC.

**Supplementary Information:**

The online version contains supplementary material available at 10.1186/s12951-023-02078-9.

## Background

Lung cancer, specifically non-small cell lung cancer (NSCLC), is the leading cause of cancer-related death worldwide [1, 2]. NSCLC is characterized by high genetic mutation rates and a diverse tumor environment, which influences the therapeutic response, patient survival as well as quality of life [3, 4]. Chemotherapy remains the most common treatment strategy for NSCLC; however, nonspecific targeting ability, poor bioavailability, severe side effects, and acquired drug resistance limit its efficacy [5, 6]. Nanocarriers, such as albumin-bound paclitaxel and liposomal doxorubicin (Dox) as well as immune checkpoint-based active targeting drug delivery, have been developed and are clinically used for improved therapeutic efficacy, but overall benefits are limited [7]. To address these challenges, multifunctional drug delivery systems have been developed based on tumor microenvironment characteristics to improve therapeutic outcomes and minimize side effects [8]. Considering the complexity and heterogeneity of the tumor microenvironment, determining an ideal therapeutic target for effective treatment remains challenging since several antigens are ubiquitously expressed on both tumor and healthy cells [9, 10]. Therefore, it is urgent and practical to analyze the NSCLC tumor microenvironment, search for an ideal therapeutic target, and design smart drug delivery systems for devising an effective strategy for highly selective and effective tumor treatment [11].

Comprehensive analysis of the NSCLC tumor ecosystem identified B7 homolog 3 protein (B7H3, also known as CD276) as a type I transmembrane protein that is broadly overexpressed by malignant cells and tumor-associated cells, but with restricted expression in normal tissues [12, 13]. B7H3, an immune checkpoint molecule belonging to the B7 immunoglobin superfamily, plays a costimulatory or coinhibitory dual role in immune responses [14, 15]. Overexpression of B7H3 in tumor cells, tumor-associated vasculature, and stromal fibroblasts contributes to tumor angiogenesis, invasion, and metastasis, and correlates with fewer tumor-infiltrating lymphocytes and poor clinical prognosis [16, 17]. Importantly, B7H3 is highly expressed in tumor-infiltrating blood vessels but not pathological angiogenesis, and thus, has higher specificity; this can facilitate specific tumor therapy since vascular endothelial growth factor therapy cannot distinguish pathological and physiological angiogenesis [18]. Although the regulation mechanisms and immune response functions of B7H3 remain nebulous, it is an attractive target for the precise treatment of NSCLC [19]. Currently, monoclonal antibodies and antibody–drug conjugates based on B7H3 have been developed and have shown a good safety profile in phase I clinical trials, thus presenting a good application prospect [20].

Photothermal therapy (PTT) is a promising alternative antitumor strategy to traditional chemotherapy to mitigate multi-drug resistance and cancer recurrence, ultimately enhancing therapeutic efficacy [21–23]. Electromagnetic navigation bronchoscopy (ENB) has been widely used in the diagnosis and therapeutics of clinical pulmonary lesions with safety and reliability which other methods cannot offer [24, 25]. ENB combined with a near-infrared (NIR) laser source is an instrument for PTT, which possesses inherent advantages including non-invasiveness, tissue penetration capability, and accelerating drug release in target sites [26, 27]. Gold nanocages (GNCs) are ideal candidate platforms for combined chemo- and photothermal therapy due to their hollow interior, porous walls, strong NIR absorption, excellent photothermal conversion efficiency, modifiability, and biocompatibility [28, 29]. Nonetheless, conventional GNCs are limited by their short retention, poor accumulation, and intratumoral penetration [30]. A smart drug delivery system that responds to cues from stimuli, such as acidic pH, GSH, and enzymes in the tumor microenvironment to achieve selective drug release at the tumor sites with fewer side effects is ideal; it should also exhibit a greatly improved retention and drug loading capacity [31, 32]. In addition, the NIR laser can be used as an external stimulus for these intelligent multifunctional gold nanocage conjugates to generate local hyperthermia and tumor ablation with minimal adverse effects on healthy tissues.

**Scheme 1:**
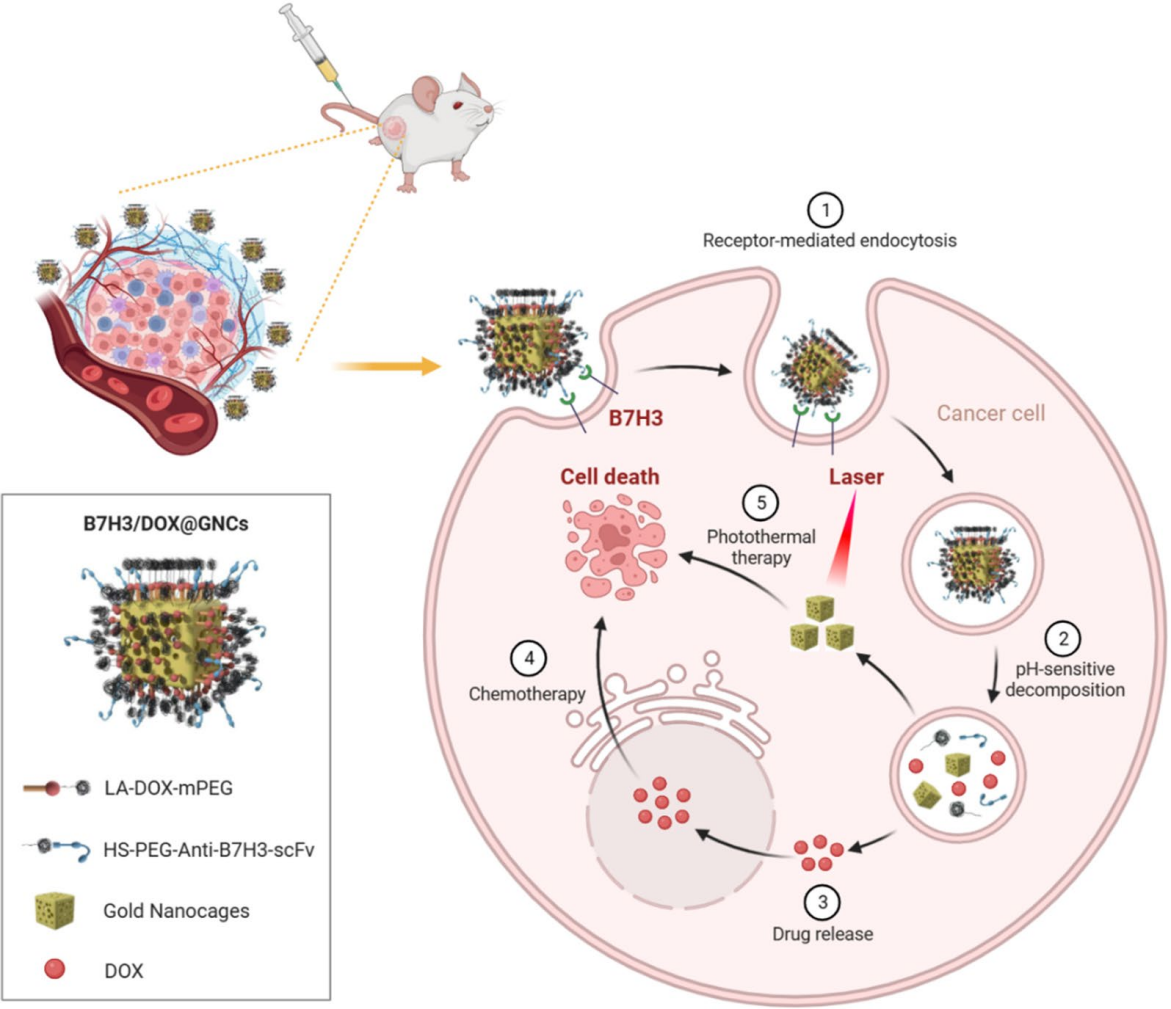
Schematic illustration of the cellular uptake and drug release of B7H3/Dox@GNCs for synergistic chemo-photothermal therapy against NSCLC

Herein, we took advantage of B7H3 as a dual target and designed a smart multifunctional gold nanocage conjugate that underwent pH-triggered drug release and exhibited efficient tumor hyperthermia ablation. GNCs, anti-B7H3 scFv, and Dox were employed as the model carrier, targeting ligand, and drug, respectively, to fabricate B7H3 targeting Dox-conjugated GNCs (B7H3/Dox@GNCs) for precise spatial–temporal synchronization of chemotherapy-photothermal therapy against NSCLC (Scheme 1). We hypothesized that: (1) B7H3/Dox@GNCs could selectively target B7H3 positive tumor cells, tumor vasculature, and tumor-associated stromal cells; (2) high stability and long retention in circulation and in situ drug release of Dox would respond to an acidic environment and esterase in tumor cells; (3) NIR-exposure could accelerate drug release and achieve precise spatial–temporal synchronization of chemotherapy/photothermal therapy with low side effects against NSCLC.

## Results

### Isolation of high-affinity anti-human B7H3 scFv

**Fig. 1:**
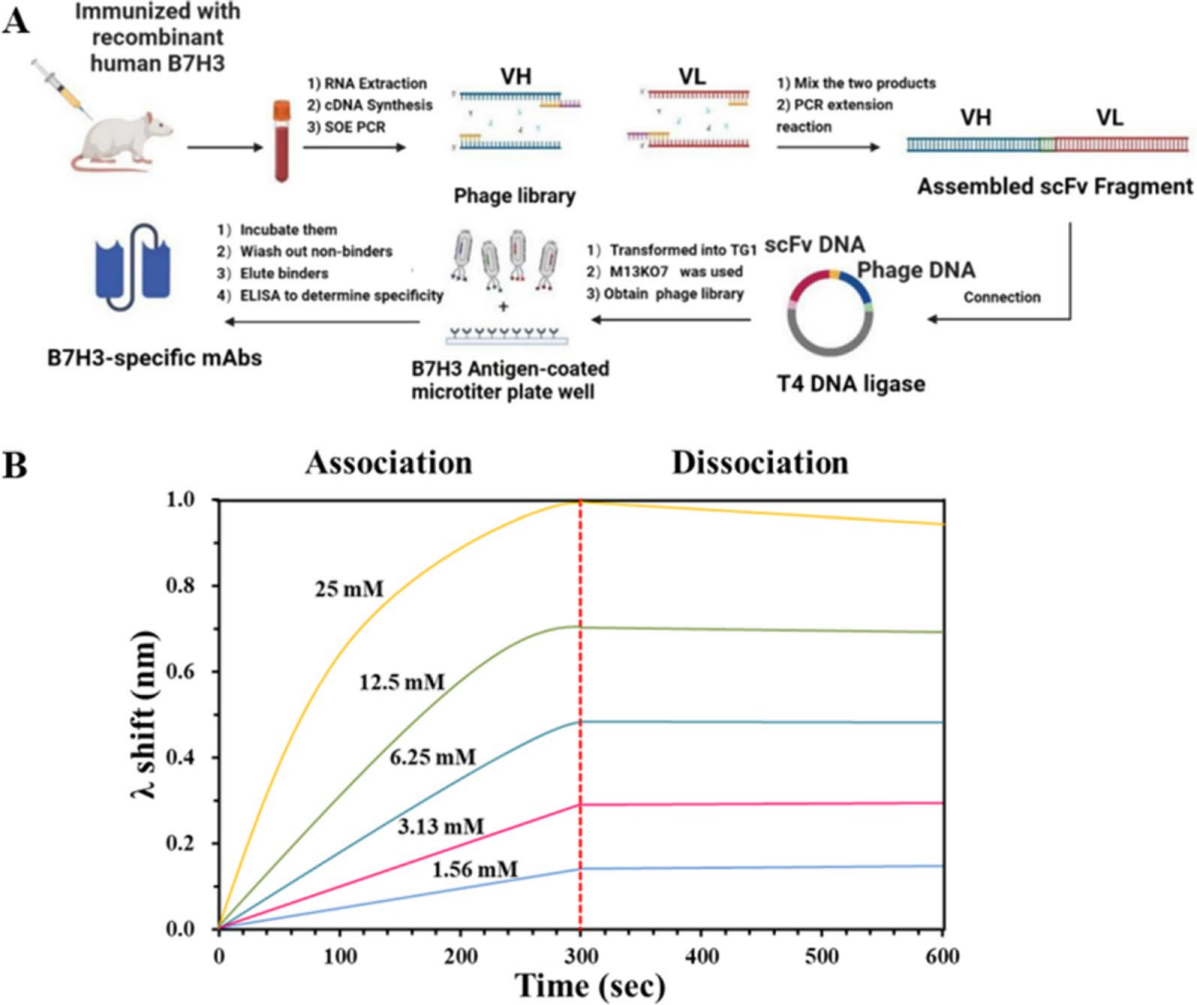
Isolation and characterization of anti-human B7H3 scFv. **A** A schematic figure of all steps to isolate high affinity anti-human B7H3 scFv. **B** BLI measurements of anti-human B7H3 against B7H3 antigen

In total, 8 heavy chain and 16 light chain mutants were constructed. The heavy and light chain vectors were paired to transfect HEK293 cells to express 128 full-length IgG candidates. After four additional rounds of selection, we obtained variants that bound B7H3 with dissociation constants (K_D_) as low as 177 pM (Fig. 1B).

### Preparation and characterization of B7H3/Dox@GNCs

**Fig. 2:**
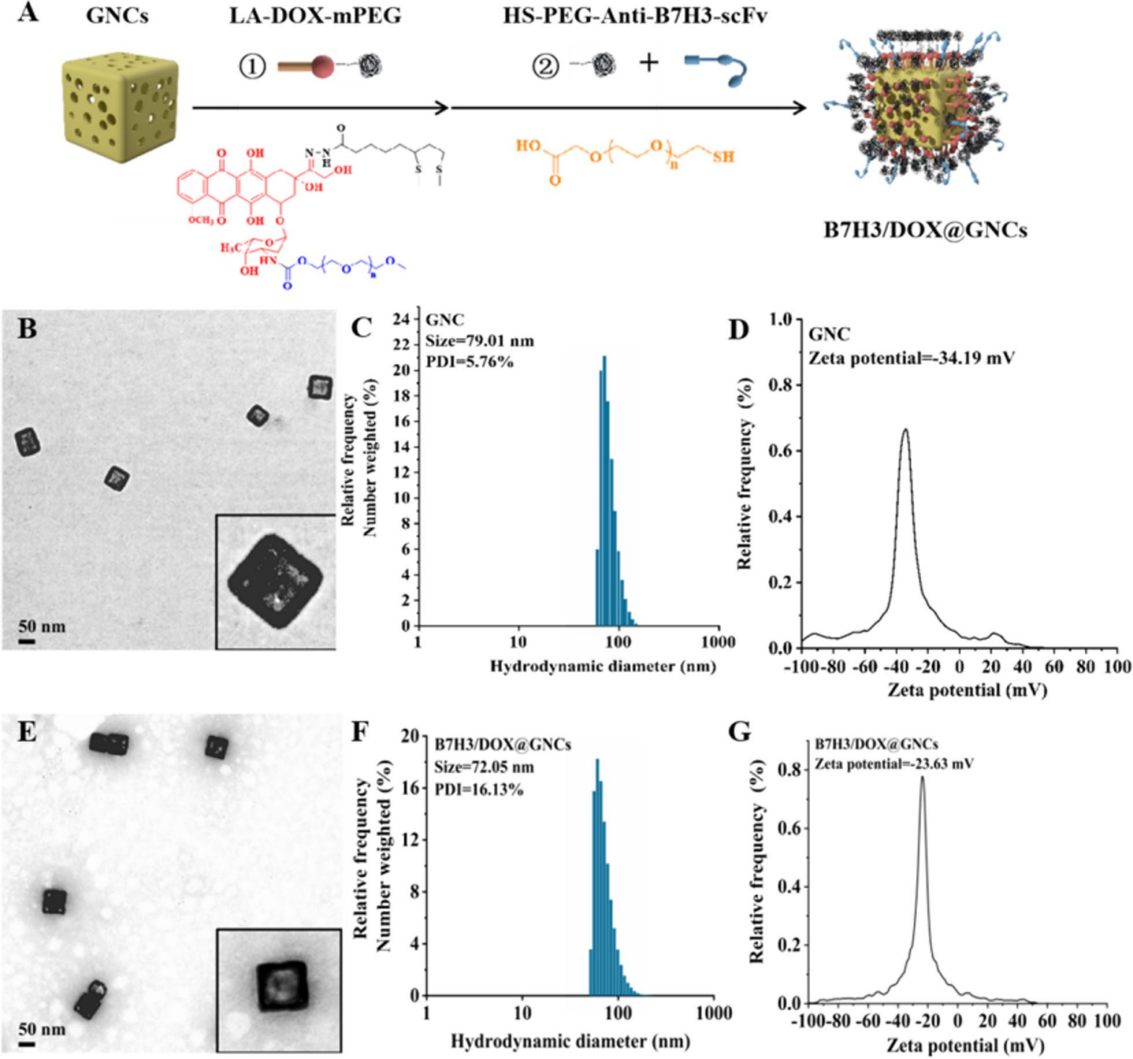
Synthesis and characterization of B7H3/Dox@GNCs. **A** The preparation and structure of B7H3/Dox@GNCs. **B** TEM image of GNCs (Scale bar, 50 nm). **C** Hydrodynamic diameters and **D** Zeta potentials of GNCs **E** TEM image of B7H3/Dox@GNCs (Scale bar, 500 nm). **D** Hydrodynamic diameters and **E** Zeta potentials of B7H3/Dox@GNCs using dynamic light scattering

B7H3/Dox@GNCs were prepared and used for combined chemotherapy/photothermal therapy targeting tumor and tumor-associated cells. The specific design principle of B7H3/Dox@GNCs is illustrated in Fig. 2A. The core was a GNC with an appropriate particle size as a carrier to deliver drugs and antibodies and a heat source for high-efficiency photothermal conversion. Dox was a hydrophilic polyethylene glycol covalently attached to the surface of the GNCs through a pH-sensitive hydrazone bond. The chemical reaction schemes used to synthesize the pH-sensitive therapeutic ligands (LA-Dox-mPEG) are presented in the Supporting Information (Additional file 1: Fig. S2). Structural characterization of the chemical intermediate and final products of LA-Dox-mPEG using ultraviolet–visible (UV–vis) spectra, FT-IR, ^13^C, and ^1^H nuclear magnetic resonance spectroscopy (NMR) is illustrated in Supporting Information (Additional file 1: Figs. S3–S5). The anti-B7H3 scFv was coupled to the bifunctional polyethylene glycol through the lysine group to generate a sulfhydryl group and connected to the surface of the gold nanocage.

The hydration diameter of the silver nanocube was approximately 60.86 nm, the polydispersity coefficient was 15.15%, and the zeta potential was − 36.92 mV. Transmission electron microscopy showed that the silver nanocubes had a regular cube shape with an approximately 49.23 nm long side, and had uniform distribution. The GNC was prepared using the gold-silver replacement method [33]. The final hydration diameter of the GNC was approximately 79.01 nm, with a polydispersity coefficient of 5.76% and good particle size distribution. The zeta potential was − 34.19 mV, and the SPR absorption peak was noted at 790 nm. As shown in Additional file 1: Fig. S1, the hydrated particle size of the synthesized B7H3/Dox@GNCs, detected via dynamic light scattering (DLS), was 72.05 nm, which was slightly smaller than that of the unmodified GNCs since the larger molecular weight PVP on the surface of GNCs was replaced by antibodies and polyethylene glycol with lower molecular weight. As shown in Fig. 2G, the zeta potential of B7H3/Dox@GNCs was − 23.63 mV, which was more neutral than that of unmodified GNCs and thus beneficial for cellular internalization; its UV–Vis spectrum showed an LSPR peak at 787 nm (Additional file 1: Fig. S1D–F), while the 808 nm laser resonated with the crystal nucleus of GNCs so that light energy could be efficiently converted into heat energy. As shown in Fig. 2B**–**E, the morphological observation of the prepared GNCs using transmission electron microscopy showed a regular cube shape and a hollow structure, with a cube edge length of approximately 65.22 nm with uniform distribution. Meanwhile, the static morphology of B7H3/Dox@GNCs showed a halo around the nanoparticles after negative staining with phosphotungstic acid, suggesting a layer of organic matter around the GNCs, which were expected to be antibodies.

**Fig. 3:**
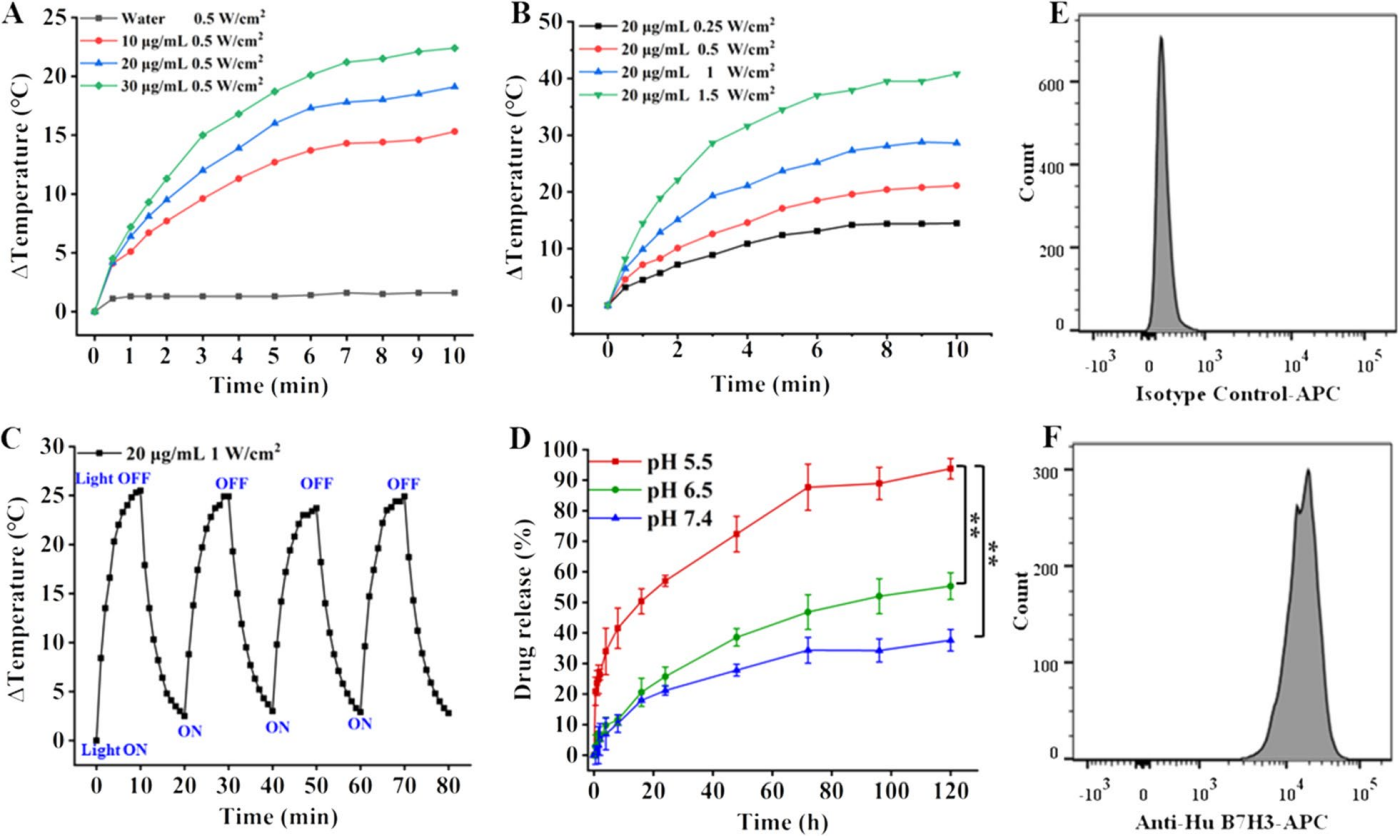
Temperature variation curve and pH-sensitive Dox release of B7H3/Dox@GNCs. **A** Temperature increasing curve of B7H3/DOX@GNCs under different solution concentrations. **B** Temperature increasing curve of B7H3/DOX@GNCs under with different NIR irradiation intensity. **C** Photothermal stability of B7H3/DOX@GNCs with four cycles of laser on/off. **D** The release curve of DOX from B7H3/DOX@GNCs at pH 7.4, 6.5, and 5.5. Data are Mean ± SD (n = 3), ^*^P < 0.05 and ^**^P < 0.01, (one-sample t-test) versus pH 5.5 group. **E**, **F**) flow cytometry quantification of B7H3 variants on NCI-H1299 cells (Top picture is the isotype control group, bottom picture is the experimental group)

As shown in Additional file 1: Fig. S1H, the loading of Dox increased with increasing concentration of the Dox derivative solution. When GNCs (C_Au_ = 10 μg/mL) were mixed with 5 nM LA-Dox-mPEG, the maximum amount of Dox connected was up to 0.35 μg/mL. When a higher concentration of LA-Dox-mPEG was added to the solution, the maximum amount of Dox connected did not significantly change, indicating that the maximum LA-Dox-mPEG connection had been achieved on the gold nanocages. The connection rate was 12.06%, while the maximum drug load was 2.22%; pH 7.4, 6.5, and 5.5 were used to mimic the lysosomal pH in normal tissues, tumor sites, and tumor cells, respectively. The plots showing the percentage of drug released from B7H3/Dox@GNCs as a function of time at different pH values (Fig. 3D) demonstrated that the release rate of Dox significantly varied with pH. At pH 7.4, the release rate of Dox was slow and the cumulative release was approximately 30% due to the stable hydrazone bond and the protective effect of mPEG, indicating good stability in the physiological environment, which would prevent nonspecific Dox release in non-tumor sites. However, at pH 6.5 and 5.5, the release rate of Dox significantly increased to 50 and 90%, respectively.

### Photothermal efficiency analysis

To explore the performance of photothermal conversion, different concentrations of B7H3/Dox@GNCs aqueous solution were exposed to NIR (0.5 W/cm^2^) for 10 min. Figure 3A shows that the temperature of the solution increased with the increase in B7H3/Dox@GNCs concentration. Specifically, the temperature of pure water increased slightly (only 1.6 ℃), whereas the temperature of 10 μg/mL B7H3/Dox@GNCs solution increased by 10 ℃ within 5 min, and after 10 min irradiation, the temperature of 30 μg/mL B7H3/Dox@GNCs solution increased by 22 ℃. Thus, B7H3/Dox@GNCs showed an effective photothermal effect.

Meanwhile, as shown in Fig. 3B, when B7H3/Dox@GNCs (C_Au_ = 20 μg/mL) was irradiated using NIR lasers of different powers, the temperature increased by more than 10 ℃ in response to as low irradiation as 0.25 W/cm^2^, while 1.5 W/cm^2^ laser irradiation increased the temperature to more than 40 ℃. The photothermal conversion efficiency of B7H3/Dox@GNCs was 20.6%, indicating its immense potential as a photothermal therapeutic agent.

### Expression of B7H3 and cell uptake analysis

To confirm the function of B7H3/Dox@GNCs inside tumor cells, their cellular uptake mechanism, cytotoxicity, and intracellular tracking were investigated. Human lung cancer cells (NCI-H1299) were used to determine B7H3 expression on the cell surface, which was as high as 99.03% (Fig. 3E**–**F) [34]. The results confirmed that NCI-H1299 cells highly expressed B7H3 protein.

**Fig. 4:**
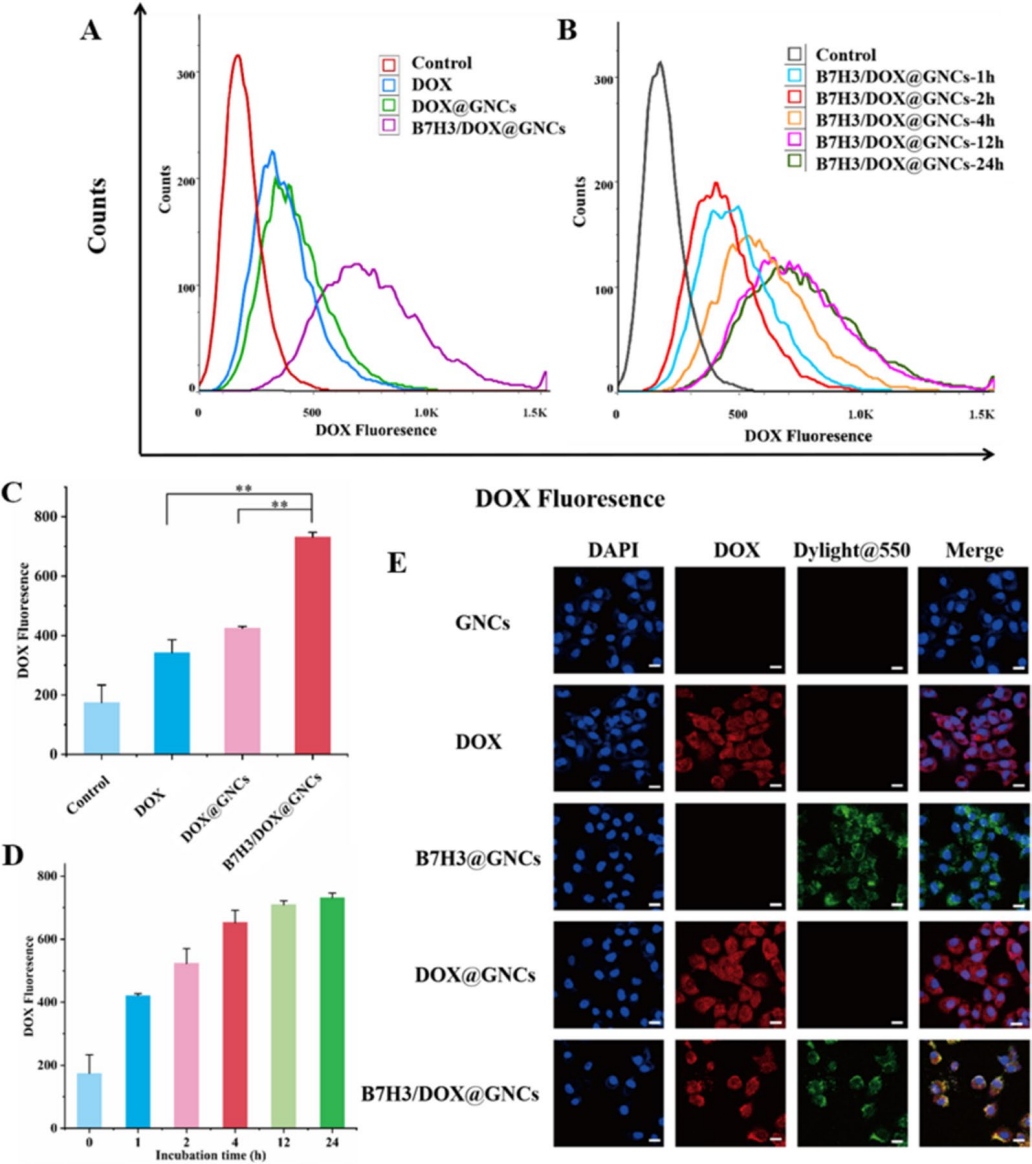
Cellular uptake and intracellular Dox release of DOX@GNCs and B7H3/DOX@GNCs in NCI-H1299 cells. **A**, **C** Flow cytometry analysis of the cellular uptakes of DOX, DOX@GNCs, and B7H3/DOX@GNCs (1 μg/mL) with incubation for 12 h. **B**, **D** Flow cytometry analysis of the cellular uptakes of B7H3/DOX@GNCs (1 μg/mL) after incubation at a different time. **E** CLSM images of cells treated with GNCs, DOX, DOX@GNCs, B7H3@GNCs, and B7H3/DOX@GNCs (1 μg/mL) for 12 h

As shown in Fig. 4A, C, after incubation with different materials for 12 h, the fluorescence intensity of Dox in cells treated with Dox@GNCs was 1.25-fold higher than that of cells treated with free Dox. The results showed that Dox could passively diffuse and permeate into the cells due to a concentration gradient. Furthermore, Dox@GNCs were endocytosed by cells due to larger particle size, and the speed of cellular uptake was lower than that of free Dox. However, upon uptake, Dox was rapidly released due to the breakage of the hydrazone bond and exerted cytotoxicity before being pumped out. These cells also showed slightly stronger fluorescence intensity than that of cells subjected to treatment with free Dox. In cells treated with B7H3/Dox@GNCs, the fluorescence intensity of Dox was significantly higher than that of free Dox (2.14-fold; p < 0.001) or Dox@GNCs (1.72-fold; p < 0.01). This indicated that the interaction between anti-B7H3-scFv and B7H3 receptor on the cell surface induced rapid cellular uptake, showing a stronger fluorescence than that of the free Dox or Dox@GNCs treatments.

Next, to determine the speed of cellular uptake, cells were treated with B7H3/Dox@GNCs for different durations (Fig. 4B, D). During the 1–12 h treatment, the Dox fluorescence intensity increased with the incubation time. When B7H3/Dox@GNCs were treated for 24 h, the Dox fluorescence intensity was only 0.05-fold higher than that at 12 h, indicating that the cells achieved maximum uptake after 12 h treatment with B7H3/Dox@GNCs.

### Intracellular tracking and drug release

To investigate intracellular tracking and drug release when different materials were used, we performed confocal laser scanning microscopy (CLSM). As shown in Fig. 4E, Dox emitted red fluorescence, Dylight ^®^550 attached to anti-B7H3-scFv emitted green fluorescence, and DAPI, used to localize the nucleus, emitted blue fluorescence.

When cells were treated with Dox, it completely diffused into the cells following a concentration gradient, and fluorescence was observed in the cytoplasm. Similarly, when cells were treated with Dox@GNCs, Dox dispersed in the cytoplasm, indicating that the cells had a good GNC@Dox uptake capacity. In the acidic lysosomal environment, Dox successfully dissociated and was released into the cytoplasm to induce cytotoxicity. The specific combination of Dylight^®^550 and anti-B7H3-scFv on B7H3/Dox@GNCs emitted green fluorescence, indicating that anti-B7H3-scFv was successfully attached to the surface of multifunctional gold nanocarriers, exhibited active targeting and specifically attached to the tumor cells, and subsequently internalized.

When cells were treated with B7H3/Dox@GNCs, a stronger red fluorescence of Dox diffused throughout the cell than in cells treated with Dox and Dox@GNCs. The red fluorescence and green fluorescence overlapped to yield yellow fluorescence, indicating that via antibody-mediated endocytosis, cells will take up more nano-drugs.

### Cytotoxicity analysis

**Fig. 5:**
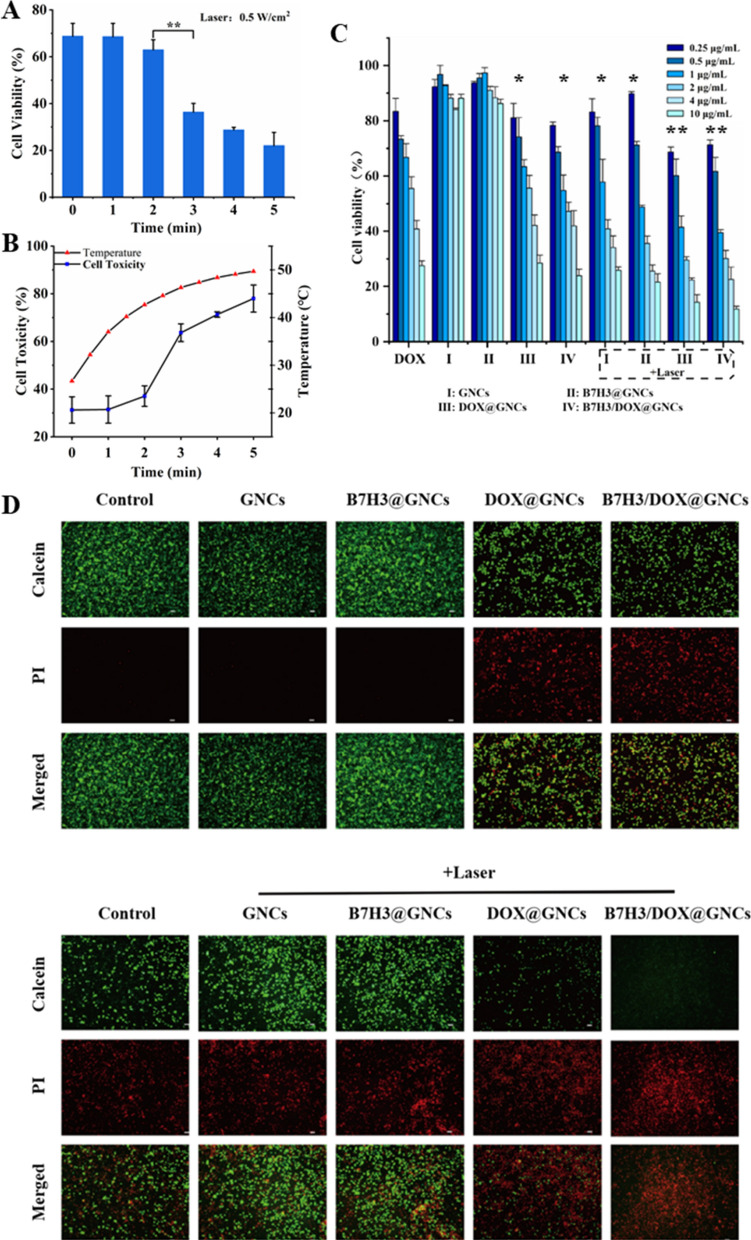
In vitro cytotoxicity of B7H3/DOX@GNCs in NCI-H1299 cells. **A**, **B** Cytotoxicity of B7H3/DOX@GNCs under with diffrent NIR irradiation time on NCI-H1299 cells for 24 h. **C** Cytotoxicity of different concentrations of GNCs, Dox, DOX@GNCs, B7H3@GNCs, and B7H3/DOX@GNCs with/without irradiation on NCI-H1299 cells for 24 h and cell viability was assessed by MTT assay. **D** Live/dead cell staining assay results of NCI-H1299 cells treated with GNCs, B7H3@GNCs, DOX@GNCs, and B7H3/DOX@GNCs with or without NIR radiation. Live cells were stained with calcein (green), and dead cells were stained with propidium iodide (red). Data are Mean ± SD (n = 3), ^*^P < 0.05 and ^**^P < 0.01, (one-sample t-test) versus GNCs group

PTT has been shown to induce cell death. As shown in Fig. 5A, B, cells treated with B7H3/Dox@GNCs (1 μg/mL Dox equivalent) showed significantly differential cytotoxicity when subjected to different durations of laser irradiation. When irradiated for 1 min, the culture medium was heated to 37 °C, and the cell survival rate was comparable to that at 0 min. When irradiated for 2 min, the culture medium was heated to 42.7 ℃, and the cell survival rate was lower than that at 0 min. However, when irradiated for 3 min, the culture medium was heated to 46.3 ℃, with a significant reduction in cell survival rate from 68.8 to 36.4% (p < 0.001). After 4 and 5 min of laser irradiation, the temperature of the culture medium reached 48.4 ℃ and 49.7 ℃, respectively, and the cell survival rate further decreased. This indicated that above 43 ℃, the cell survival rate reduced significantly. Therefore, irradiation with 0.5 W/cm^2^ laser for 3 min was selected for PTT.

Cells were treated with Dox, Dox@GNCs, and B7H3/Dox@GNCs for 24 h, and the results are shown in Fig. 5C. Without irradiation, the cytotoxicity of GNCs and B7H3@GNCs was comparable to that observed in the blank control group, and the cell survival rate remained > 80% at different concentrations. The survival rate of cells treated with Dox, Dox@GNCs, and B7H3/Dox@GNCs changed in a dose-dependent manner, with IC_50_ values of 2.50, 2.49, and 1.75 μg/mL, respectively. These results indicated that B7H3/Dox@GNCs possessed a stronger cytotoxic effect due to B7H3 receptor-mediated endocytosis. When cells were exposed to NIR laser irradiation, their survival rate decreased significantly compared to the non-irradiated group. The higher the GNC content in the culture medium, the stronger the cytotoxicity. Among different groups, the one subjected to B7H3/Dox@GNCs combined with NIR laser irradiation had notably inhibited cell proliferation, with IC_50_ reaching 0.76 μg/mL. Dysregulated signal transduction resulting from cell membrane damage, cytoplasmic protein degeneration, suppressed DNA repair and replication, and hyperthermia suppressed cell proliferation and induced cell death.

The antitumor effect of B7H3/Dox@GNCs was further verified using calcein-AM and propidium iodide (PI) staining (Fig. 5D). The blank control group, GNCs, or anti-B7H3-scFv@GNC treated cells showed strong green fluorescence, indicating that cell viability was not affected, while cells treated with GNCs or B7H3@GNCs combined with NIR laser irradiation showed strong red fluorescence, indicating a significant decrease in cell viability. The cells treated with Dox, Dox@GNCs, and B7H3/Dox@GNCs had higher counts of dead cells (strong red fluorescence). Cells treated with B7H3/Dox@GNCs showed a significant increase in cell death following NIR laser treatment, compared to that observed in Dox@GNCs or the Dox treatment group. This result confirmed that B7H3/Dox@GNCs combined with NIR laser were a suitable candidate for effective antitumor therapy.

### Effect of tumor cell cycle and reactive oxygen species levels

**Fig. 6:**
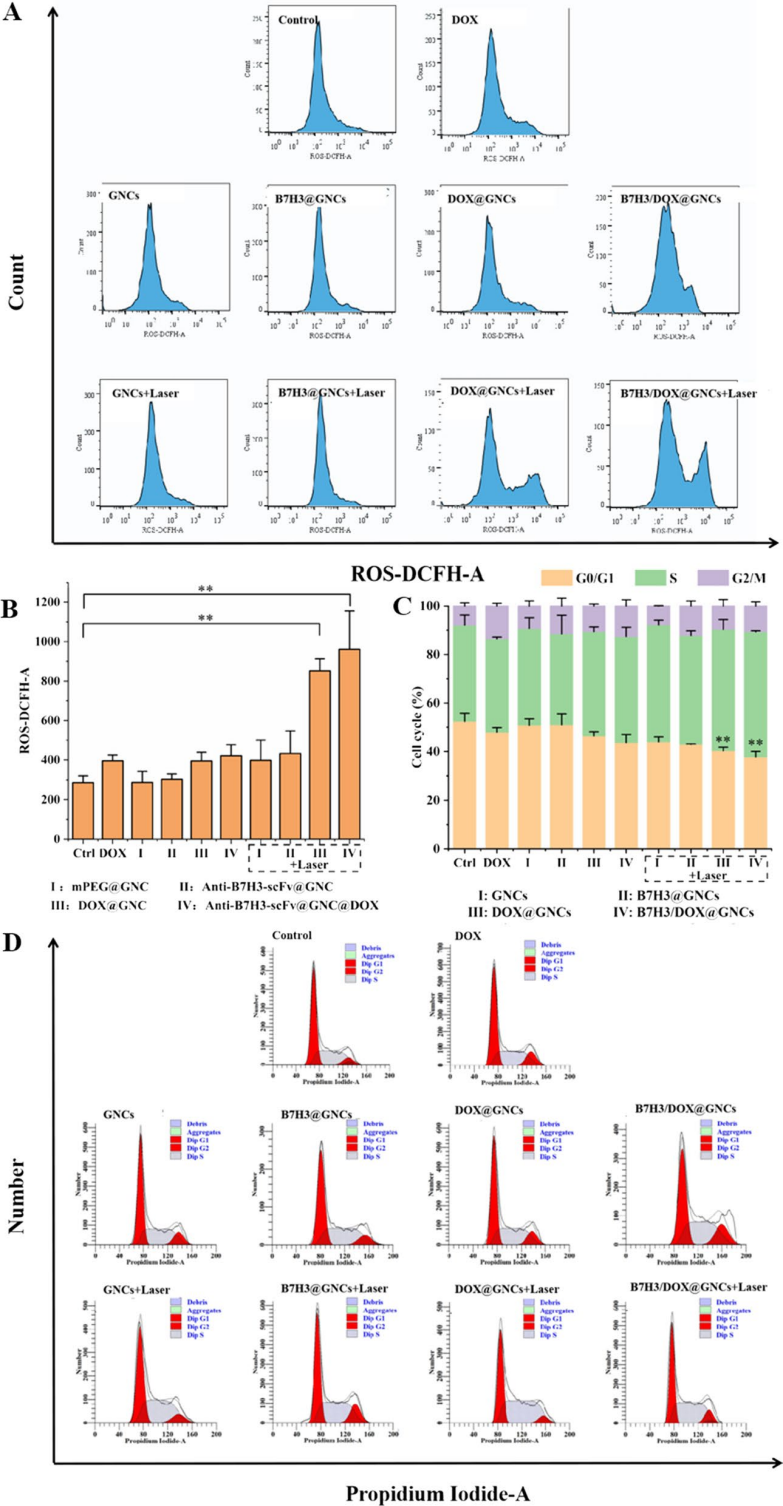
Effects of B7H3/DOX@GNCs on cell cycle and intracellular ROS generation in NCI-H1299 cells. **A**, **B** FACS analysis of intracellular ROS generation of cells treated with GNCs, B7H3@GNCs, DOX@GNCs, and B7H3/DOX@GNCs for 24 h with or without NIR radiation. **C**, **D** FACS analysis of cell cycle distributions of cells treated with GNCs, B7H3@GNCs, DOX@GNCs, and B7H3/DOX@GNCs for 24 h with or without NIR radiation

The cell cycle was detected using the PI/RNase method (Fig. 6C). The first peak in the cycle represents the cells with diploid DNA content in the G0/G1 phase, the second peak represents the cells with tetraploid DNA content in the G2/M phase, and the part between the two peaks indicates the cells in the S phase. As shown in Fig. 6D, cells treated with Dox, Dox@GNCs, and B7H3/Dox@GNCs without irradiation stagnated in the S phase and G2/M phase; similarly, the proportion of cells in the G0/G1 phase decreased significantly due to cell cycle arrest in the G2/M phase caused by free Dox and the interaction between Dox and DNA released from nanomaterials. Facilitation by anti-B7H3-scFv-guided ligand-receptor interaction led to more Dox being absorbed by cells and undergoing cell proliferation arrest. However, no significant change in the cell cycle was observed upon treatment with GNCs and B7H3@GNCs, indicating that GNCs and B7H3@GNCs did not affect the cell cycle.

In contrast, when cells were treated with GNCs and B7H3@GNCs, their DNA was denatured following PTT, and cells were arrested in the S and G2/M phases. In addition, when cells were treated with B7H3/Dox@GNCs plus NIR irradiation, the arrest in the S and G2/M phases was stronger than that observed following Dox treatment (the proportion of cells in G0/G1 phase significantly decreased by 10.33%, P < 0.05). This indicated that the mechanism underlying PTT-induced cell cycle arrest (S and G2/M phase) differed from that induced by Dox alone, and enhanced cell sensitivity to Dox.

DCFH-DA, a green fluorescent probe, can detect reactive oxygen species (ROS) production. Under normal circumstances, cells produce a small amount of ROS. However, Dox and high temperatures induce changes in ROS levels. As shown in Fig. 6A, B, without irradiation, GNCs- and B7H3@GNCs-treated cells produced active oxygen species compared to those produced in the blank control group. However, following NIR irradiation, cells treated with GNCs and B7H3@GNCs exhibited increased production of ROS (1.2–1.6-fold higher; P < 0.001). In the absence of NIR irradiation, ROS generation increased slightly in cells treated with Dox, Dox@GNCs, and B7H3/Dox@GNCs alone, compared with the blank control group. In the groups treated with Dox@GNCs and B7H3/Dox@GNCs combined with NIR irradiation, the intracellular ROS level was 2.15-fold (p < 0.001) and 2.43-fold (p < 0.001) higher, respectively, than that observed in the Dox group.

### In vivo pharmacokinetic and antitumor efficacy analyses

#### Pharmacokinetic and biodistribution analyses

**Fig. 7:**
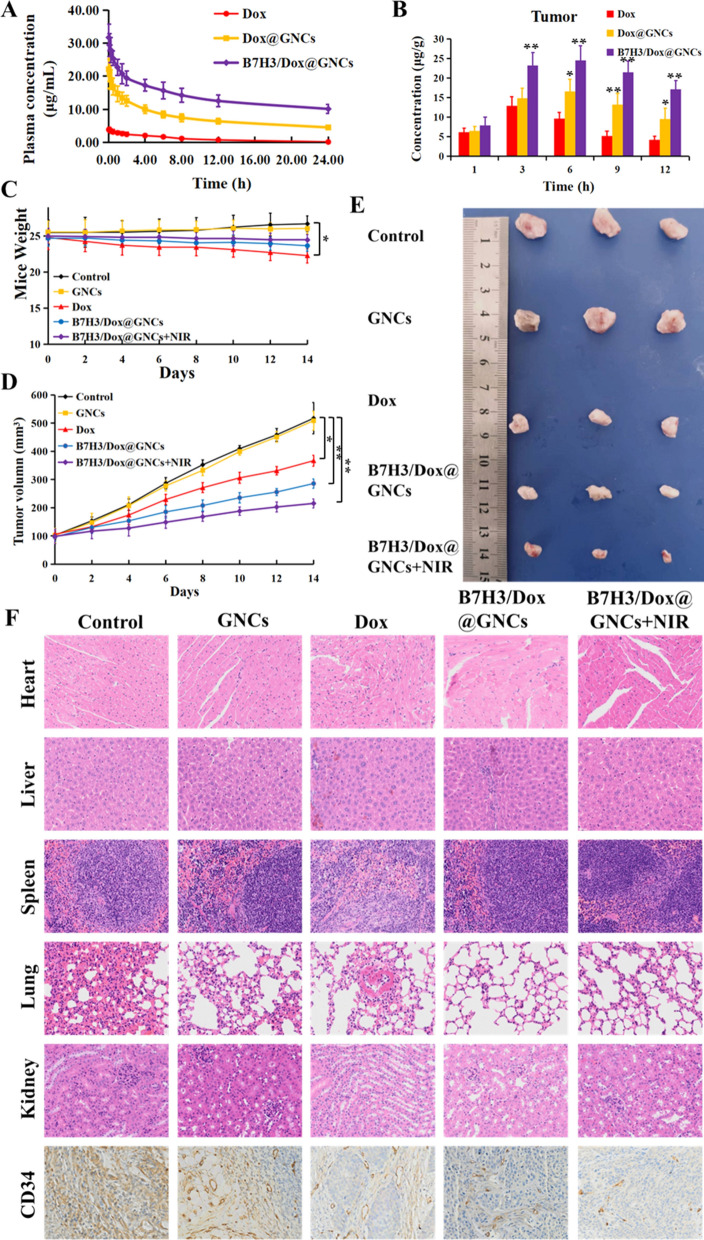
In vivo pharmacokinetic, biodistribution, and antitumor efficacy. **A** Plasma concentration–time profiles of Dox in rats following *i.v.* administration of Dox•HCl, Dox@GNCs, and B7H3/Dox@GNCs at a Dox dose of 3 mg/kg. **B** Biodistribution profiles of Dox in tumor tissues of NSCLCs tumor-bearing mice following intravenous injection of Dox·HCl, Dox@GNCs, and B7H3/Dox@GNCs at Dox dose of 3 mg/kg for different time. **C** Body weight changes, **D** Tumor volume changes, and **E** Tumor tissue photo of saline, GNCs, Dox•HCl, B7H3/Dox@GNCs and B7H3/Dox@GNCs with NIR irradiation of tumor-bearing mice during the 14 day treatment or at the 14th day. **F** H&E and CD34 sained of the the tissues including heart, liver, spleen, lung, kidney, and tumor from NSCLCs xenograft-bearing mice after intravenous administration of saline, GNCs, Dox•HCl, B7H3/Dox@GNCs and B7H3/Dox@GNCs with NIR irradiation. Data are Mean ± SD (n = 3), ^*^P < 0.05 and ^**^P < 0.01, (one-sample t-test) versus Dox group

A comparative pharmacokinetic analysis was carried out to evaluate the commercial formulation of Dox•HCl, as well as Dox@GNCs and B7H3/Dox@GNCs, and the Dox concentrations of the indicated formulation in biological samples were determined using liquid chromatography-mass spectrometry (LC–MS/MS). The mean plasma Dox concentration–time curves following a single *i.v*. administration of the indicated formulations at a Dox equivalent dose of 3 mg/kg are shown in Fig. 7A. The main pharmacokinetic parameters of Dox•HCl, Dox@GNCs, and B7H3/Dox@GNCs are listed in Table 1. Both Dox@GNCs and B7H3/Dox@GNCs showed higher Dox plasma concentrations than Dox•HCl, indicating a higher plasma protein binding rate and faster elimination of Dox from blood circulation. These results demonstrate Dox@GNCs and B7H3/Dox@GNCs have a longer blood circulation time and better stability in circulation, due to the protective effect of the mPEG shell that prevents their rapid clearance and reduces the non-target absorption. Comparison of the pharmacokinetic parameters between Dox@GNCs, B7H3/Dox@GNCs, and Dox•HCl showed that the area under the plasma concentration–time curve (AUC_0-∞_) for Dox@GNCs and B7H3/Dox@GNCs was 6.73 and 15.58-fold higher, respectively than that of the control. This indicates that both conjugates exhibited good stability and sustained, controlled release with a long circulation time. In addition, B7H3/Dox@GNCs showed the lowest clearance rate (CL, 0.05 ± 0.00 L/h) and apparent volume of distribution (Vd, 0.87 ± 0.56 L) values compared to Dox•HCl and Dox@GNCs, indicating the slowest elimination rate of Dox in vivo. At the same time, B7H3/Dox@GNCs displayed the highest plasma half-life (T_1/2_, 21.32 ± 2.46 h) and mean residence time (MRT_0-∞_, 23.48 ± 2.06 h) among the tested formulations, confirming a longer circulation characteristic and suggesting that the surface modification with anti-B7H3 scFv may facilitate longer retention in the tumor.

**Table 1:** Pharmacokinetic parameters of Dox•HCl, Dox@GNCs, and B7H3/Dox@GNCs after single intravenous administration in rats (Mean ± SD, n = 6)

PK Parameters	Dox•HCl	Dox@GNCs	B7H3/Dox@GNCs
T_1/2_ (h)	8.67 ± 0.34	14.72 ± 1.36^**^	21.32 ± 2.46^**^
AUC_0-∞_ (h·mg/mL)	36.49 ± 1.27	245.48 ± 17.92^**^	568.45 ± 36.54^**^
V_d_ (L/kg)	1.64 ± 0.41	1.15 ± 0.63^**^	0.87 ± 0.56^**^
CL (L/h/kg)	0.13 ± 0.00	0.08 ± 0.00^**^	0.05 ± 0.00^**^
MRT_0-∞_(h)	9.43 ± 0.46	15.97 ± 1.54^**^	23.48 ± 2.06^**^

^*^P < 0.05, ^**^P < 0.01 versus Dox•HCl group (one-sample t-test)

The biodistribution profiles of the indicated Dox formulations in rats are shown in Fig. 7B and Additional file 1: Fig. S6. Retention and accumulation of Dox in non-target tissues are attributed to major side effects of Dox formulations, particularly cardiotoxicity [35]. After *i.v.* administration, the Dox concentrations were significantly higher in the heart, liver, lungs, spleen, and kidneys of the Dox•HCl group compared to those in the Dox@GNCs and B7H3/Dox@GNCs groups, indicating that the gold conjugates exhibited lower retention, reduced toxicity, and faster elimination than Dox•HCl. These data confirm that free Dox was widely distributed and rapidly eliminated in all the tested tissues. In addition, the tumor tissue Dox concentration in the B7H3/Dox@GNCs group was much higher than those in Dox•HCl and Dox@GNCs groups at all-time points after *i.v.* administration.

#### Antitumor efficacy study

The efficacy of B7H3/Dox@GNCs as a lung tumor targeting agent and its synergistic chemo/PTT effect was evaluated using a mouse model of NCI-H1299 cell-derived xenograft, as shown in Fig. 7C–E. In mice treated with saline and GNCs without Dox, the tumors rapidly grew during the experiment. The administration of Dox•HCl resulted in suppressed tumor growth but also resulted in a drastic decrease in body weight and a significant decrease in the quality of life of the mice. After a 2 week treatment interval with B7H3/Dox@GNCs and NIR irradiation, the tumor volume decreased by 0.42-, 0.58-, and 0.75-fold compared to tumors of mice treated with saline, Dox•HCl, and B7H3/Dox@GNCs, respectively. This significant suppression can be attributed to the effective targeting and sustained drug release via B7H3 targeting and pH-sensitive release and was supported by the results of pharmacokinetic studies. The group subjected to NIR laser irradiation after *i.v.* administration of B7H3/Dox@GNCs showed the smallest tumor volume and strongest apoptosis, likely due to the homotypic accumulation induced by B7H3 targeting and the significant hyperthermia. Furthermore, the body weight of mice treated with B7H3/Dox@GNCs was comparable to the control groups, indicating improved quality of life with low systemic toxicity.

To comparatively evaluate the impact of the conjugates on normal tissues, tumors, and tumor vasculature, a histological examination was performed (Fig. 7F). Compared to the disorganized arrangement and disrupted myocardial fibers, as well as edema and vacuolization observed in the heart tissue sections of other groups, Dox-induced damage to the heart was observed only in the Dox•HCl group, indicating that the conjugates showed fewer side effects associated with Dox. IHC staining of the NCI-H1299 lung tumor showed that the vessel density in the tumors was significantly reduced in the B7H3/Dox@GNCs group.

## Discussion

In the present study, we used high-affinity anti-B7H3 scFv and Dox-PEG derivatives as surface-functionalized ligands to GNCs and simultaneously destroyed both B7H3-positive tumor vasculature and tumor cells. Under acidic conditions, the hydrazone bond was protonated and rapidly cleaved, thereby releasing Dox into the medium. B7H3/Dox@GNCs efficiently and rapidly entered tumor cells by antibody-mediated endocytosis and achieved maximum uptake after 12 h co-incubation with NCI-H1299 cells, which is 20 times higher than free Dox. In a word, B7H3/Dox@GNCs would concentrate at the tumor site or be taken up into the tumor cell, releasing Dox and exerting cytotoxic effects.

Moreover, B7H3/Dox@GNCs displayed remarkable photothermal stability (Fig. 3C) since the temperature rise did not change significantly even after four cycles of irradiations, which indicated B7H3/Dox@GNCs had a good NIR photothermal conversion effect and excellent photothermal stability [36]. Tumor cells were treated with a combination of PTT and chemotherapy exhibited elevated ROS levels, which negatively impacted their physiological state. These in vitro results indicated that B7H3/Dox@GNCs combined with laser irradiation had a stronger tumor-killing effect than that of Dox alone.

B7H3/Dox@GNCs effectively enhanced the drug's stability and prevented its rapid clearance from the body. The longer drug half-life (T_1/2_) and mean residence time (MRT_0-∞_) of the smart nanocage conjugates benefit from providing protection against enzymatic degradation and improving its circulation time and tumor-specific accumulation. In addition, pharmacodynamics studies results suggested that higher amounts of Dox were delivered to tumor sites when anti-B7H3 scFv modified pH-sensitive gold nanocage conjugates were used, suggesting that higher accumulation and sustained, controlled release in the tumor will have improved therapeutic efficacy with fewer side effects [37]. All in all, the precise control over the order and duration of drug exposure, both spatially and temporally, at the desired site led to improved therapeutic efficacy through chemotherapy/photothermal therapy.

## Conclusions

The multifunctional gold nanocarrier (B7H3/Dox@GNCs) targeted tumor cells, tumor-associated vasculature, and stromal fibroblasts overexpressing B7H3 cell-surface protein and delivered Dox with excellent stability, dispersibility, and biocompatibility. GNCs exhibited an efficient photothermal conversion effect. The multifunctional gold nanocarrier had an ideal hydrated size (72.05 nm) and a near-neutral zeta potential (− 23.63 mV), resulting in a slow elimination rate in circulation. Anti-B7H3-scFv can be attached to the surface of GNCs via LA-PEG-COOH, thereby enhancing biological targeting. After chemical structure modification, Dox was sulfated to conjugate on the surface of GNCs and PEGylated to generate a hydration layer. Our conjugate was not only highly stable in circulation but also exhibited pH-responsive and selective drug release in tumor cells.

Compared with free Dox, multifunctional gold conjugates showed improved antitumor efficacy, precise accumulation into tumors, and B7H3 antibody receptor-mediated effective endocytosis. Under NIR irradiation and acidic stimuli, the enhanced hyperthermia ablation of multifunctional GNCs with minimum undesired effects, together with spatial and temporal order and duration of drug exposure achieved synergistic therapy of NSCLC via combined chemo- and photothermal therapy. In addition, the strategy of synchronous treatment destroyed B7H3 positive tumor cells, tumor-associated vasculature, and stromal fibroblasts through synergistic chemotherapy-PTT, in combination with fiberoptic bronchoscope to achieve non-invasive local phototherapy, which provides great potential for theranostics in future clinical translations [38].

## Material and methods

### Materials

Lipoic acid, chloroauric acid trihydrate, Sulfo-NHS, HS-PEG-COOH, and mPEG-SH 1-ethyl-(3-dimethylaminopropyl)carbodiimide hydrochloride (EDC), were purchased from Shanghai Aladdin Chemical Reagent Co., Ltd., China; polyvinylpyrrolidone (PVP-K30) and silver nitrate were purchased from Sinopharm Chemical Reagent Co., Ltd.; doxorubicin hydrochloride (Dox) was purchased from Dalian Meilun Biotechnology Co., Ltd.; RPMI-1640 modified liquid medium was purchased from HyClone; DAPI, Calcein/PI cell viability and cytotoxicity detection kits, reactive oxygen species detection kits, cell cycle and apoptosis detection kits, and The death detection kit were purchased from Shanghai Biyuntian Biotechnology Co., Ltd.; Dylight^®^550 was purchased from Abbott (Shanghai) Trading Co., Ltd.

### Isolation and characterization of anti-human B7H3 scFv

#### Animals

All animal experiments were approved by the Experimental Animal Center of Fujian Medical University. Animal experiments were carried out in compliance with the Guide for the Animals Care and Ethics Committee of Fujian Medical University (Certificate number: FJMU IACUC 2021-0339). All animals were housed in a pathogen-free facility with an ambient temperature of 25 ± 2 ℃, relative humidity of 55 ± 5%, and had free access to water and food.

#### Isolation of mouse anti-human B7H3 antibody

To obtain a high-affinity B7H3 targeting antibody, we screened and characterized recombinant human single-chain variable fragment (scFv) against B7H3 from a naïve human antibody library [39]. We created mutant libraries of phage particles displaying scFv generated following immunization with recombinant human B7H3; the scFvs were produced via transformation of electrocompetent TG1 cells with an scFv sequence harboring vector. Three 8 week female BALB/c mice were immunized with recombinant human B7H3 (NCBI NO. 80381) protein. After booster immunization, their spleens were harvested and the lysate was prepared. Total RNA was extracted from spleen cell lysate using TRIzol and reverse-transcribed to cDNA. PCR was performed to amplify heavy chain and light chain variable regions using mouse-specific primers, and products were assembled into an scFv library via slicing by overlap extension PCR.

The scFv DNA and phagemid DNA were cut separately with the restriction enzymes, before being ligated together with T4 DNA ligase. Next, the ligation mixture was desalted and electro-transformed into competent TG1 cells. M13KO7 helper phage was used to rescue and obtain a phage display scFv library. The library was used to isolate specific scFv binders to B7H3 via affinity panning. Human B7H3 antigen was used to screen specifically bound antibodies through four consecutive panning steps. The binding ability of selected monoclonal phage clones was tested using phage ELISA. Positive clones were selected, sequenced, and phylogenetically analyzed. Based on phylogenetic distance and sequence diversity, representative antibodies were selected for transient expression, purification, and characterization.

#### Humanization of mouse anti-human B7H3 antibody

The sequence of the selected scFv sequence was humanized to reduce immunogenicity. Briefly, a homology search was conducted to determine the best human V_H_ and V_L_ acceptor sequence, murine complementarity-determining regions (CDRs, donor) was grafted to the acceptor framework region to form a humanized canonical sequence, and critical amino acid residues supporting CDR loop structure were back-mutated. Heavy chain and light chain mutants were formatted to full-length IgG vectors and transiently expressed. The supernatants were subjected to ELISA to test antibody expression and antigen binding.

#### Expression and purification of scFv and Fc-containing antibody

pcDNA3.1( +) vector was used to construct all recombinant expression vectors. His tag was inserted into the C-terminal of selected scFv and subcloned into pcDNA3.1( +). For the scFv-Fc antibody, the scFv sequence was fused to the N-terminal of human IgG1 Fc. For full-length antibodies, heavy chains and light chains were separately constructed. Expi293 cells (Gibco) were transfected according to the manufacturer’s instructions. Cells induced for transient expression were cultured for approximately 12 days in a chemically defined, serum-free OPM-293 CD03 medium (OPM Biosciences, Shanghai, China) supplemented with 2 mM L-glutamine. OPM-CHO PFF05 (Shanghai OPM Biosciences) was added as feed and glucose was added as needed. For scFv, harvested supernatant was purified using the HisTrap FF column (Cytiva, Washington, USA). For the Fc-containing antibody, the HiTrap MabSelect SuRe column (Cytiva, Washington, USA) was used. Eluted antibody was buffer exchanged with PBS (pH 7.4) using a Sephadex G-25 desalting column. Bio-layer interferometry was used to assess antibody affinity. Briefly, human B7-H3 antigen was loaded onto a streptavidin sensor, then an antibody was added to monitor the binding signal.

### Preparation of B7H3/Dox@GNCs

#### Synthesis of gold nanocages

GNCs were prepared as previously described. For synthesizing AgNCs, 6 mL ethylene glycol was added to a round-bottomed flask and heated at 155 ℃ for 1 h, following which 100 μL of 3 mM Na_2_S solution was added; 8 min later, 1.5 mL of 20 mg/mL polyvinyl pyrrolidone glycol solution was added to the solution and finally, 1 min later, 0.5 mL of 48 mg/mL AgNO_3_ glycol solution was added. During the reaction, a series of color changes was observed till a milky yellow color finally appeared.

For the synthesis of GNCs, to a 5 mL 1 mg/mL PVP aqueous solution, 100 μL silver cube solution was added and stirred at 100 ℃ at 300 rpm for 10 min. Via a dropwise method, 0.1 mM HAuCl_4_ solution was added; the LSPR absorption wavelength of the solution was detected using a microplate reader, and the reaction was terminated when the maximum absorption wavelength of the reaction solution reached near 800 nm.

#### Synthesis of pH-responsive LA-Dox-mPEG.

To achieve retention and tumor-specific triggered drug release, we designed and synthesized a thiol-terminated Dox-PEG derivative for selective acidic stimulus drug release, following previous reports [31, 32]. For synthesizing LAOEt in the dark under a nitrogen atmosphere, lipoic acid, ethanol, and p-dimethylamino pyridine were dissolved in dichloromethane, and N, N'- dicyclohexylcarbodiimide was dissolved in dichloromethane. This solution mixture was added dropwise at 0 ℃, stirred for 1 h, and heated to 25 ℃ for 24 h to continue the reaction. Next, the solution was filtered, and the filtrate was concentrated and separated using column chromatography (petroleum ether: ethyl acetate = 15:1) to obtain yellow oily ethyl LAOEt. For the synthesis of LA-NHNH2, LAOEt was dissolved in anhydrous methanol under nitrogen protection and dark conditions, stirred at 40 ℃, and ethyl acetate was added dropwise to the dilute solution, extracted with saturated sodium chloride solution, and the organic layer was dried with anhydrous magnesium sulfate for 8 h, centrifuged, and the supernatant was concentrated and separated using column chromatography (dichloromethane: methanol = 15:1) to obtain light yellow solid lipoacyl trap LA-NHNH2. For the synthesis of LA-Dox, LA-NHNH2, and Dox were dissolved in anhydrous methanol under nitrogen protection and dark conditions, trifluoroacetic acid was added dropwise, stirred at 25 ℃ for 8 h, concentrated to 2 mL, transferred to 25 mL acetonitrile, underwent ultrasonic treatment for 5 min, incubated for 8 h at 4 ℃, centrifuged, washed with acetonitrile thrice, and dried to obtain deep red powder doxorubicin lipoylate LA-Dox. For the synthesis of LA-Dox-mPEG, LA-Dox, and mPEG-NPC were dissolved in anhydrous dimethylformamide at 25 °C under nitrogen protection and dark conditions. TEA was added to the solution, and after stirring for 24 h, the solution was steamed at 30 ℃ to remove the solvent, separated, purified using column chromatography (dichloromethane: methanol = 50:1–15:1), and lyophilized to obtain red solid LA-Dox-mPEG.

#### Synthesis of B7H3/Dox@GNCs

Firstly, a lipoic acid-modified Dox-mPEG (LA-Dox-mPEG) was incorporated into the framework of GNCs [40]. Then, thiol-terminated anti-B7H3 scFv targeting ligands with high affinity, as the shells and protein corona, were coated to the surface of GNCs. HS-PEG-COOH with a final concentration of 0.5 nM was added to GNCs (C_Au_ = 30 μg/mL) while stirring at 25 °C for 4 h. mPEG-SH with a final concentration of 100 nmol/mL was added later while stirring at 25 °C for an additional 4 h; a 25 nM of EDC and Sulfo-NHS was added while stirring at 25 °C for 0.5 h. The reagent was centrifuged to remove the supernatant, and resuspended in deionized water; 0.05 nM of Anti-B7H3-scFv single chain antibody was added while stirring for 4 h, centrifuged to remove the supernatant, and finally resuspended in an equal volume of deionized water to obtain B7H3@GNCs. For the synthesis of GNCs, mPEG-SH with a final concentration of 100 nM was added to GNCs (C_Au_ = 30 μg/mL) while stirring at 25 °C for 4 h, centrifuged to remove the supernatant, and resuspended in an equal volume of deionized water to obtain GNCs. For the synthesis of Dox@GNCs, mPEG-SH with a final concentration of 0.5 nM was added to GNCs (C_Au_ = 30 μg/mL) while stirring at 25 °C for 4 h. LA-Dox-mPEG with a final concentration of 15 nM was reduced with 1.5 times equivalent of NaBH4 while stirring for 1 h at 0 ℃ and added to the reaction solution at 25 °C for an additional 4 h. The reaction solution was centrifuged to remove the supernatant and resuspended in an equal volume of deionized water to obtain Dox@GNCs. For synthesizing B7H3/Dox@GNCs, HS-PEG-COOH (final concentration: 0.5 nM) was added to GNCs (C_Au_ = 30 μg/mL) while stirring at 25 °C for 4 h. LA-Dox-mPEG (final concentration: 15 Nm) was reduced with a 1.5-fold equivalent amount of NaBH_4_ while stirring for 1 h at 0 ℃. This was added to the reaction solution and incubated at 25 °C for an additional 4 h. Next, 25 nM EDC and Sulfo-NHS were added while stirring at 25 °C for 0.5 h. The mixture was centrifuged to remove the supernatant and the pellet was resuspended in deionized water. Finally, 0.05 nM anti-B7H3-scFv single-chain antibody was added to the solution while stirring for 4 h, centrifuged to remove the supernatant, and the pellet was resuspended in an equal volume of deionized water to obtain B7H3/Dox@GNCs [41].

### Characterization of GNCs, LA-Dox-mPEG, and B7H3/Dox@GNCs

The particle size and zeta potential of nanoparticles were analyzed using DLS, while the morphology was analyzed via transmission electron microscopy. The gold content in GNCs was detected via an inductively coupled plasma emission spectrometer, and the purity of synthesized LA-Dox-mPEG was characterized using Fourier transform infrared spectroscopy, LC–MS, NMR, and high-performance liquid chromatography. Simultaneously, the biological stability of B7H3/Dox@GNCs stored for various durations was investigated using DLS.

LA-Dox-mPEG with final concentrations of 0.5, 1, 2, 5, 10, and 20 nM were obtained following reduction with NaBH_4_ and addition to GNCs (CAu = 10 μg/mL). The GNCs were centrifuged and resuspended in an equal volume of 0.2 M hydrochloric acid solution for 24 h. To study the potential Dox loading capacity, B7H3/Dox@GNCs were dissolved in a strong acid buffer to ensure complete dissociation of Dox. The supernatant was collected via centrifugation, and the content of Dox in the supernatant was measured via fluorescence spectrophotometry. The release behavior of B7H3/Dox@GNCs in vitro was investigated using the dialysis method. An accurately measured 1 mL solution of B7H3/Dox@GNCs in a dialysis bag (cut-off molecular weight = 3500 Da) was added to an EP tube (pH 7.4, 6.5, and 5.5) containing 10 mL release media with different pH values; three samples at each pH were set along with the thermostatic oscillator at 37 ℃ and 100 rpm. We sampled 400 μL of the media at scheduled time intervals (0, 0.5, 1, 1.5, 2, 4, 8, 16, 24, 48, 72, 96, and 120 h), supplemented the same volume of release medium, determined the concentration of Dox via fluorescence spectrophotometry, and calculated the cumulative release amount.

### Photothermal efficiency analysis

#### Photothermal detection of different gold concentrations

B7H3/Dox@GNCs dispersions (C_Au_ = 10, 20, and 30 μg/mL) were irradiated for 10 min with an 808 nm laser at a power density of 0.5 W/cm^2^. The temperature was measured using a temperature probe immersed in the dispersions every 30 s. The influence of laser power on photothermal effect was evaluated as follows: B7H3/Dox@GNCs dispersions (C_Au_ = 10 μg/mL) were irradiated for 10 min with 808 nm laser at power densities of 0.25, 0.5, 1, and 1.5 W/cm^2^. The temperature was measured using a temperature probe immersed in the dispersions every 30 s.

#### Photothermal stability test

B7H3/Dox@GNCs (C_Au_ = 20 μg/mL) was irradiated for 10 min with an 808 nm laser at a power density of 1 W/cm^2^ and cooled at 25 °C for another 10 min. The irradiation-cooling cycles were repeated four times and the temperature was measured using a temperature probe immersed in dispersions every 30 s.

### Expression of B7H3 and cell uptake analysis

Approximately 10^6^ cells of NCI-H1299 cell suspension were added to a flow tube along with 5 μL Anti-Hu B7H3-APC to one group, isotype control Mouse IgG Kappa Isotype Control-APC to the other group, and flow buffer was added at 4 ℃ for 30 min in the dark; FACS buffer was used to wash twice. Finally, the cell pellet was resuspended in a staining buffer, and the expression of B7H3 on the cell surface was measured using flow cytometry.

The cellular uptake of multifunctional gold nanoparticles was evaluated using flow cytometry and CLSM. Briefly, we incubated NCI-H1299 cells (1 × 10^5^ cells/mL) with Dox, Dox@GNCs, B7H3@GNCs, or B7H3/Dox@GNCs (1 μg/mL Dox equivalent) for 24 h and stained the samples with Dylight^®^550 and DAPI. The results were observed with CLSM and quantitative analysis was conducted using flow cytometry.

### Cytotoxicity analysis in vitro

The cytotoxicity of B7H3/Dox@GNCs on tumor cells was assessed using the MTT assay. Briefly, NCI-H1299 cells (4 × 10^3^ cells/well) were seeded in a 96-well plate and incubated for 12 h until the cells were completely attached. Different concentrations of Dox, GNCs, B7H3@GNCs, Dox@GNCs, and B7H3/Dox@GNCs (0, 0.25, 0.5, 1, 2, and 4 μg/mL equivalents) were added followed by photothermal treatment (808 nm laser, 3 min, 0.5 W/cm^2^). The cells were cultured for another 12 h and cell viability was determined using the MTT method.

### Effect of tumor cell cycle and reactive oxygen species levels

Briefly, NCI-H1299 cells (5 × 105 cells/mL) were cultured for 12 h to allow complete adherence. Next, Dox, GNCs, B7H3@GNCs, Dox@GNCs, or B7H3/Dox@GNCs (1 μg/mL Dox equivalent) were added to the culture medium. The laser irradiation group was irradiated with 808 nm (0.5 W/cm^2^) for 3 min and cultured for an additional 24 h. After the cells were fixed, RNase A solution was added to degrade RNA, and cells were stained with PI. Finally, the samples were subjected to cell cycle analysis using flow cytometry. ROS was detected using the method described above, except that the cells were stained with DCFH-DA and analyzed using flow cytometry.

### In vivo pharmacokinetic and antitumor efficacy studies

#### Pharmacokinetic and biodistribution studies

The pharmacokinetic properties of B7H3/Dox@GNCs were studied using 18 male Sprague–Dawley rats, weighing 200 ± 20 g. The rats were randomly divided into three groups (n = 6 per group) and administered Dox•HCl, Dox@GNCs, and B7H3/Dox@GNCs (3 mg/kg Dox equivalent dose) intravenously (*i.v.*) through the tail vein. Next, 300 μL blood samples were collected into heparinized tubes from the venous plexus at 0, 0.05, 0.13, 0.25, 0.5, 1, 1.5, 2, 4, 6, 8, 12, and 24 h. After centrifugation at 3000 × *g* for 15 min, the plasma samples were obtained and stored at – 80 ℃ until analysis.

A xenograft model was established using BALB/c athymic nude male mice (weight: 20 ± 2 g) and NCI-H1299 cells. Once tumors grew to approximately 200 mm^3^ in size, the mice were randomly divided into Dox·HCl, Dox@GNCs, and B7H3/Dox@GNCs groups (3 mg/kg Dox equivalent dose) and these agents were *i.v.* administered through the tail vein. The mice were sacrificed and tissues (including the heart, liver, lungs, spleens, kidneys, and tumors) were harvested at 1, 3, 6, 9, and 12 h (n = 3). The collected tissues were rinsed in ice-cold physiological saline to remove the superficial blood and other debris and stored at − 80 ℃ until analysis.

The collected tissues were diced into small pieces and homogenized with three volumes of saline. Subsequently, 100 μL plasma and tissue homogenate samples were spiked with 10 μL epirubicin as the internal standard and vortex-mixed with 300 μL methanol for 5 min. After 15 min of centrifugation at 15,000 × *g* and 4 °C to precipitate proteins, 5 μL supernatant aliquots were analyzed using HPLC–MS/MS.

#### Antitumor efficacy study

The heterotopic xenograft mouse model of NSCLC was established by subcutaneous injection of 1.0 × 10^7^ NCI-H1299 cells into the right dorsal side of BALB/c nude mice. The tumor size and body weight of the mice were measured and recorded every 2 days. The tumor volume (mm^3^) was calculated using the formula: V = W^2^ × L/2, where the width (W) and length (L) were measured using an electronic caliper. When the tumor volume reached 80–100 mm^3^, mice were administered *i.v.* Dox at a dose of 3 mg/kg in the indicated formulation at 3 day intervals via the tail vein until euthanized.

### Statistical analysis

All data were presented as Mean ± standard deviation (SD). Independent t-tests were conducted to evaluate the differences between the groups. Statistical significance was defined as ^*^P < 0.05 indicating a significant difference, and ^**^P < 0.01 indicating a highly significant difference.

### Supplementary Information

**Additional file 1: Figure S1.** Synthesis and characterization of silver nanocubes and gold nanocages. A) The photo, B) Hydrodynamic diameters, and C) Zeta potentials of silver nanocubes. D) The UV-Vis spectra of silver nanocubes, E) gold nanocages, and F) B7H3/Dox@GNCs. G) The photo, H) Hydrodynamic diameters, and I) Zeta potentials of gold nanocages. **Figure S2.** The synthetic procedures of LA-Dox-mPEG. **Figure S3.** Characterization of LAOEt and LA-NHNH2. A) HPLC chromatograms, B) FT-IR, C) MS spectra, G) 1H-NMR (DMSO-d6) and H) 13C-NMR (DMSO-d6) of LAOEt. D) HPLC chromatograms, E) FT-IR, F) MS spectra, H) 1H-NMR (DMSO-d6) and J) 13C-NMR (DMSO-d6) of LA-NHNH2. **Figure S4.** Characterization of LA-Dox and mPEG-NPC. A) HPLC chromatograms, B) FT-IR, C) MS spectra, G) 1H-NMR (DMSO-d6) and H) 13C-NMR (DMSO-d6) of LA-Dox. D) HPLC chromatograms, E) FT-IR, F) MS spectra, H) 1H-NMR (DMSO-d6) and J) 13C-NMR (DMSO-d6) of mPEG-NPC. **Figure S5.** Characterization of LA-Dox-mPEG. A) HPLC chromatograms, B) FT-IR, C) MS spectra, D) 1H-NMR (DMSO-d6) of LA-Dox-mPEG. **Figure S6.** In vivo biodistribution of Dox, Dox@GNCs, and B7H3/Dox@GNCs. B) Biodistribution profiles of Dox in A) heart, B) Lung, C) Liver, D) Spleen, and E) Kindey of NSCLCs tumor-bearing mice following intravenous injection of Dox·HCl, Dox@GNCs, and B7H3/Dox@GNCs at Dox dose of 3 mg/kg for different time. Data are Mean ± SD (n=3), *P < 0.05 and **P < 0.01, (one-sample t-test) versus Dox group.

## Data Availability

The data set that support the findings of this study are available from the corresponding author on reasonable request.

## Abbreviations

B7-H3: B7 homolog 3 protein
CDR: Complementarity-determining region
CLSM: Confocal laser scanning microscopy
DLS: Dynamic light scattering
Dox: Doxorubicin hydrochloride
Dox@GNC: Doxorubicin-conjugated gold nanocage
EDC: 1-Ethyl-(3-dimethylaminopropyl)carbodiimide hydrochloride
ENB: Electromagnetic navigation bronchoscopy
GNC: Gold nanocage
IHC: Immunohistochemistry
LC–MS: Liquid chromatography-mass spectrometry
MRT: Mean residence time
NIR: Near-infrared
NMR: Nuclear magnetic resonance
NSCLC: Non-small-cell lung cancer
PI: Propidium iodide
PTT: Photothermal therapy
scFv: Single-chain variable fragment
UV–Vis: Ultraviolet–visible
