# Supplementary material for: B7H3 targeting gold nanocage pH-sensitive conjugates for precise and synergistic chemo-photothermal therapy against NSCLC
Source: J Nanobiotechnology. 2023 Oct 17;21:378. doi: 10.1186/s12951-023-02078-9 (PMC10583352; doi:10.1186/s12951-023-02078-9)
Supplement: Supplementary file 1 — Additional file 1: Figure S1. Synthesis and characterization of silver nanocubes and gold nanocages. A) The photo, B) Hydrodynamic diameters, and C) Zeta potentials of silver nanocubes. D) The UV-Vis spectra of silver nanocubes, E) gold nanocages, and F) B7H3/Dox@GNCs. G) The photo, H) Hydrodynamic diameters, and I) Zeta potentials of gold nanocages. Figure S2. The synthetic procedures of LA-Dox-mPEG. Figure S3. Characterization of LAOEt and LA-NHNH2. A) HPLC chromatograms, B) FT-IR, C) MS spectra, G) 1H-NMR (DMSO-d6) and H) 13C-NMR (DMSO-d6) of LAOEt. D) HPLC chromatograms, E) FT-IR, F) MS spectra, H) 1H-NMR (DMSO-d6) and J) 13C-NMR (DMSO-d6) of LA-NHNH2. Figure S4. Characterization of LA-Dox and mPEG-NPC. A) HPLC chromatograms, B) FT-IR, C) MS spectra, G) 1H-NMR (DMSO-d6) and H) 13C-NMR (DMSO-d6) of LA-Dox. D) HPLC chromatograms, E) FT-IR, F) MS spectra, H) 1H-NMR (DMSO-d6) and J) 13C-NMR (DMSO-d6) of mPEG-NPC. Figure S5. Characterization of LA-Dox-mPEG. A) HPLC chromatograms, B) FT-IR, C) MS spectra, D) 1H-NMR (DMSO-d6) of LA-Dox-mPEG. Figure S6. In vivo biodistribution of Dox, Dox@GNCs, and B7H3/Dox@GNCs. B) Biodistribution profiles of Dox in A) heart, B) Lung, C) Liver, D) Spleen, and E) Kindey of NSCLCs tumor-bearing mice following intravenous injection of Dox·HCl, Dox@GNCs, and B7H3/Dox@GNCs at Dox dose of 3 mg/kg for different time. Data are Mean ± SD (n=3), *P < 0.05 and **P < 0.01, (one-sample t-test) versus Dox group. [file 12951_2023_2078_MOESM1_ESM.docx]

**Supporting Information**

**B7H3 Targeting Gold Nanocage pH-Sensitive Conjugates for Precise and Synergistic Chemo-Photothermal Therapy against NSCLC**

Bing Chen ^1,2†^, Kaifan Zheng ^1,2†^, Shubin Fang ^1,2†^, Kangping Huang ^1^, Chengchao Chu ^4^, Junyang Zhuang ^1,2^, Jin Lin ^2^, Shaoguang Li ^1,2^, Hong Yao ^1,2^, Ailin Liu ^1,2^, Gang Liu ^4*^, Jizhen Lin ^1,3,5*^, Xinhua Lin ^1,2*^

^1^ Key Laboratory of Nanomedical Technology (Education Department of Fujian Province), School of Pharmacy, Fujian Medical University, Fuzhou 350122, China.

^2^ Department of Pharmaceutical Analysis, School of Pharmacy, Fujian Medical University, Fuzhou 350122, China.

^3^ The Cancer Center, Union Hospital, Fujian Medical University, Fuzhou 350122, China.

^4^ State Key Laboratory of Molecular Vaccinology and Molecular Diagnostics & Center for Molecular Imaging and Translational Medicine, School of Public Health, Xiamen University, Xiamen 361102, China.

^5^ The Department of Otolaryngology, Head and Neck Surgery, University of Minnesota Medical School, Minneapolis 55404, United States.

^*^Correspondence: 13906939638@163.com; linjizhen@fimu.edu.cn; Gangliu.cmitm@xmu.edu.cn.

^†^ These authors contributed equally to this work.

**Keywords:** B7H3/CD276, Gold nanocage, Doxorubicin conjugates, Chemo-photothermal therapy, NSCLC


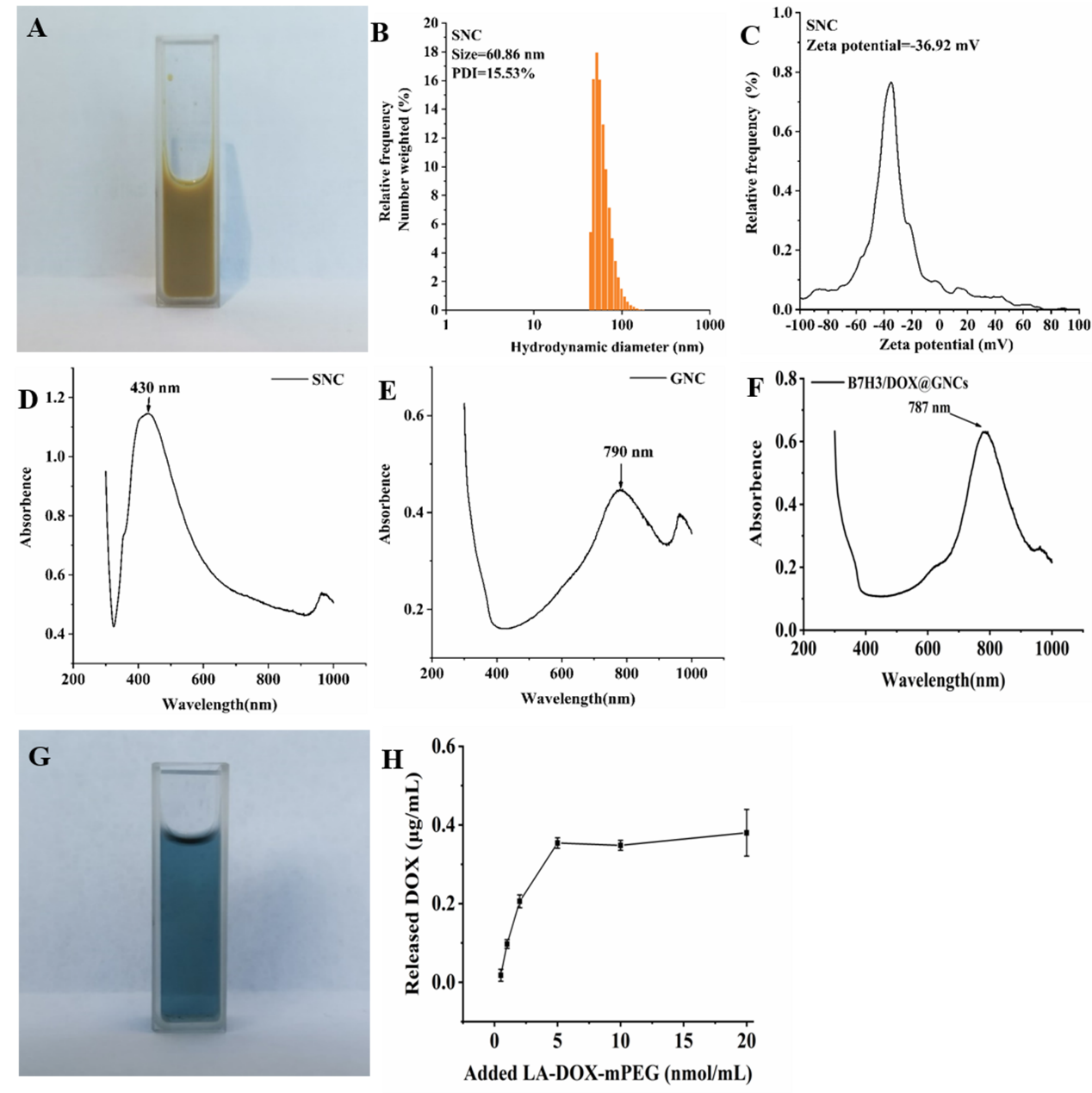


**Figure S1.** Synthesis and characterization of silver nanocubes and gold nanocages. A) The photo, B) Hydrodynamic diameters, and C) Zeta potentials of silver nanocubes. D) The UV-Vis spectra of silver nanocubes, E) gold nanocages, and F) B7H3/Dox@GNCs. G) The photo, H) Hydrodynamic diameters, and I) Zeta potentials of gold nanocages.


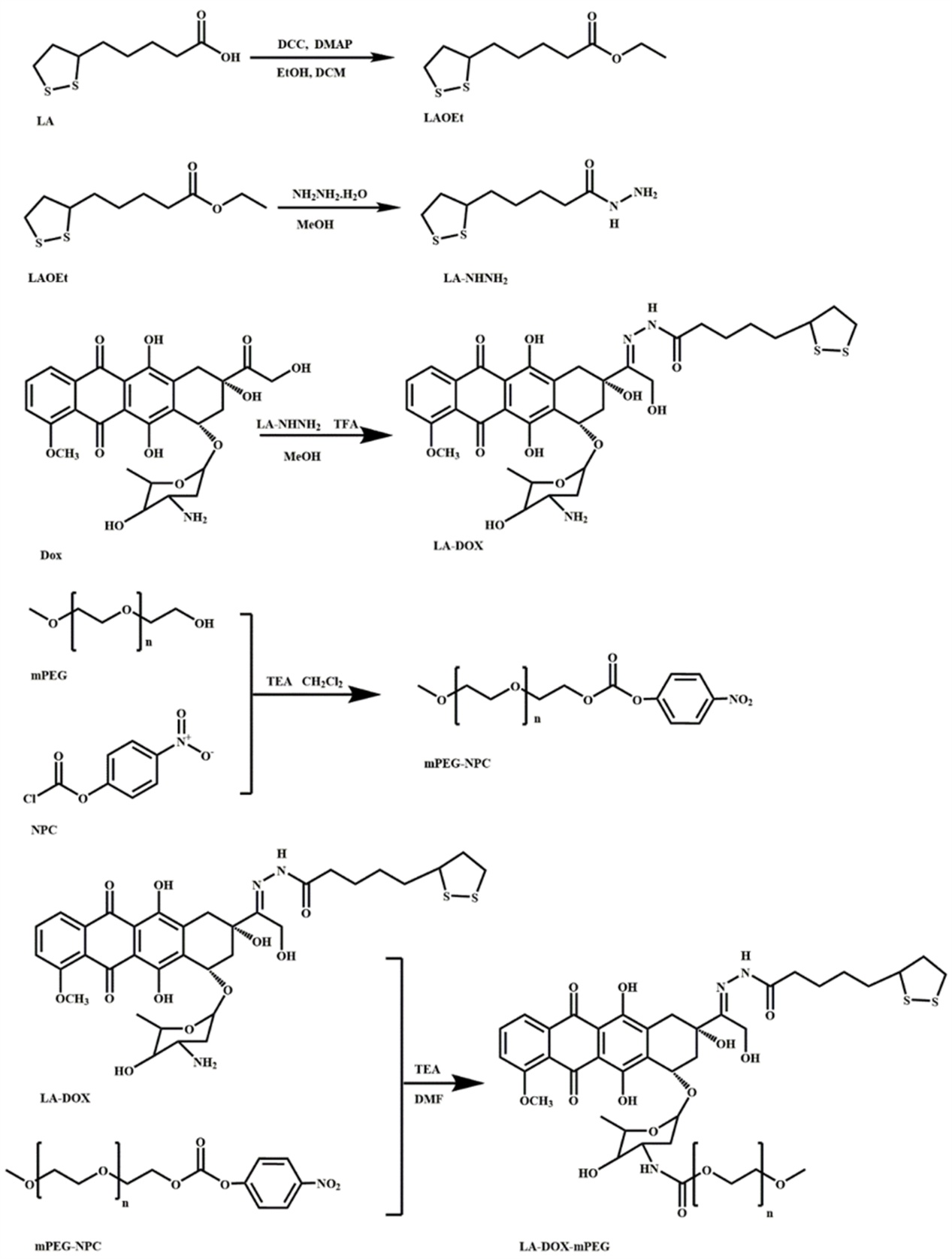


**Figure S2.** The synthetic procedures of LA-Dox-mPEG.


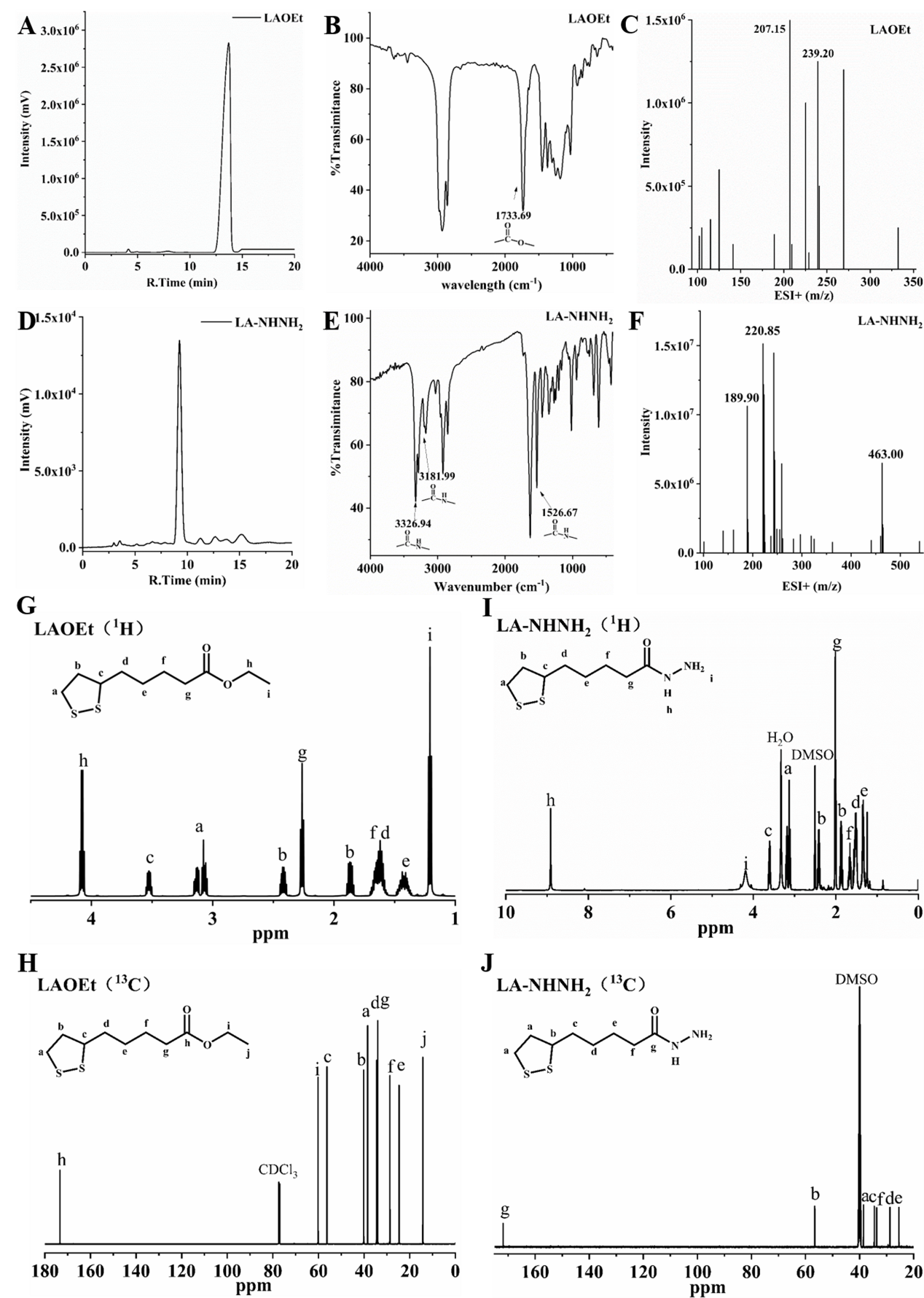


**Figure S3.** Characterization of LAOEt and LA-NHNH_2_. A) HPLC chromatograms, B) FT-IR, C) MS spectra, G) ^1^H-NMR (DMSO-d_6_) and H) ^13^C-NMR (DMSO-d_6_) of LAOEt. D) HPLC chromatograms, E) FT-IR, F) MS spectra, H) ^1^H-NMR (DMSO-d_6_) and J) ^13^C-NMR (DMSO-d_6_) of LA-NHNH_2_.


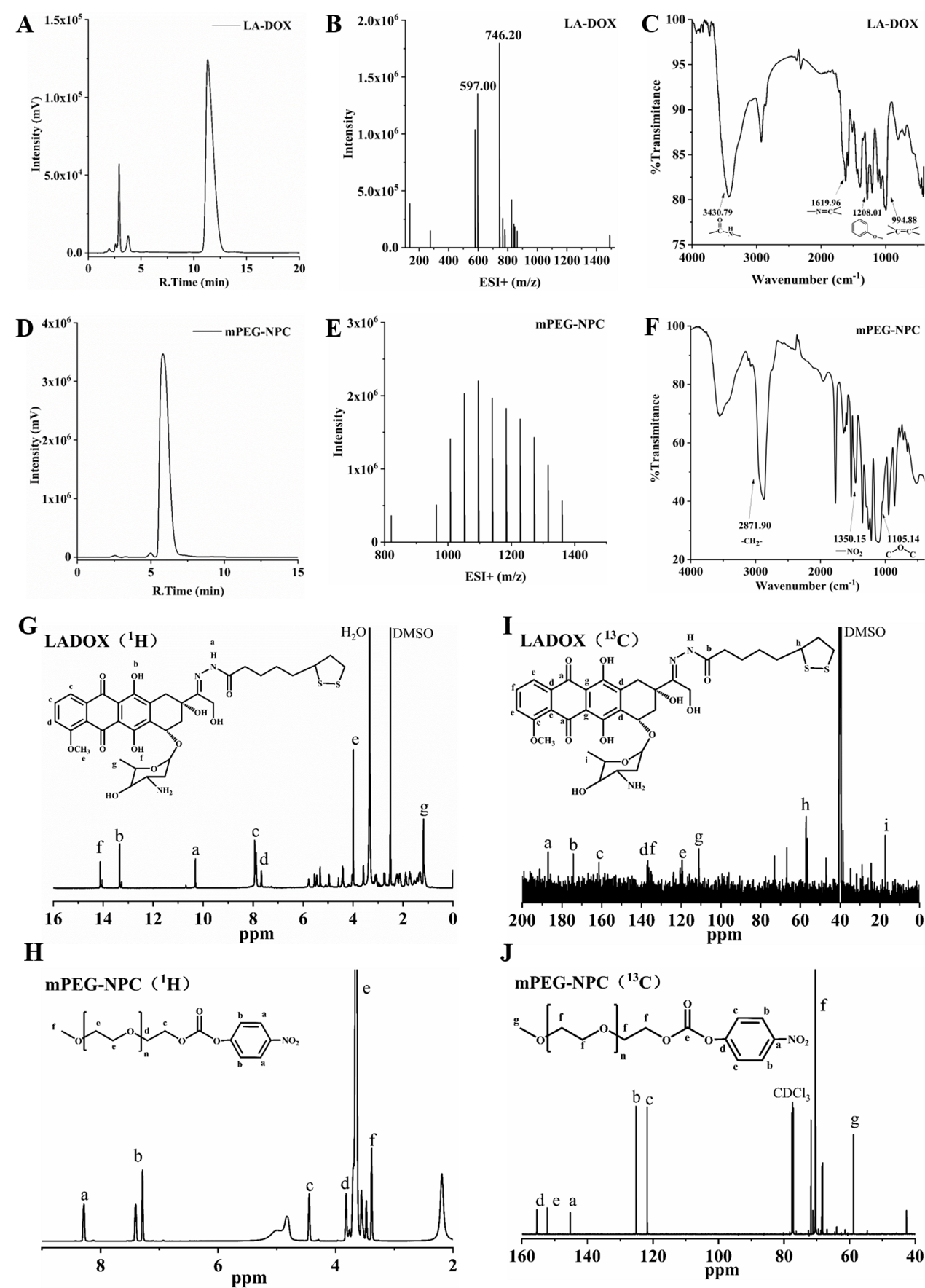


**Figure S4.** Characterization of LA-Dox and mPEG-NPC. A) HPLC chromatograms, B) FT-IR, C) MS spectra, G) ^1^H-NMR (DMSO-d_6_) and H) ^13^C-NMR (DMSO-d_6_) of LA-Dox. D) HPLC chromatograms, E) FT-IR, F) MS spectra, H) ^1^H-NMR (DMSO-d_6_) and J) ^13^C-NMR (DMSO-d_6_) of mPEG-NPC.


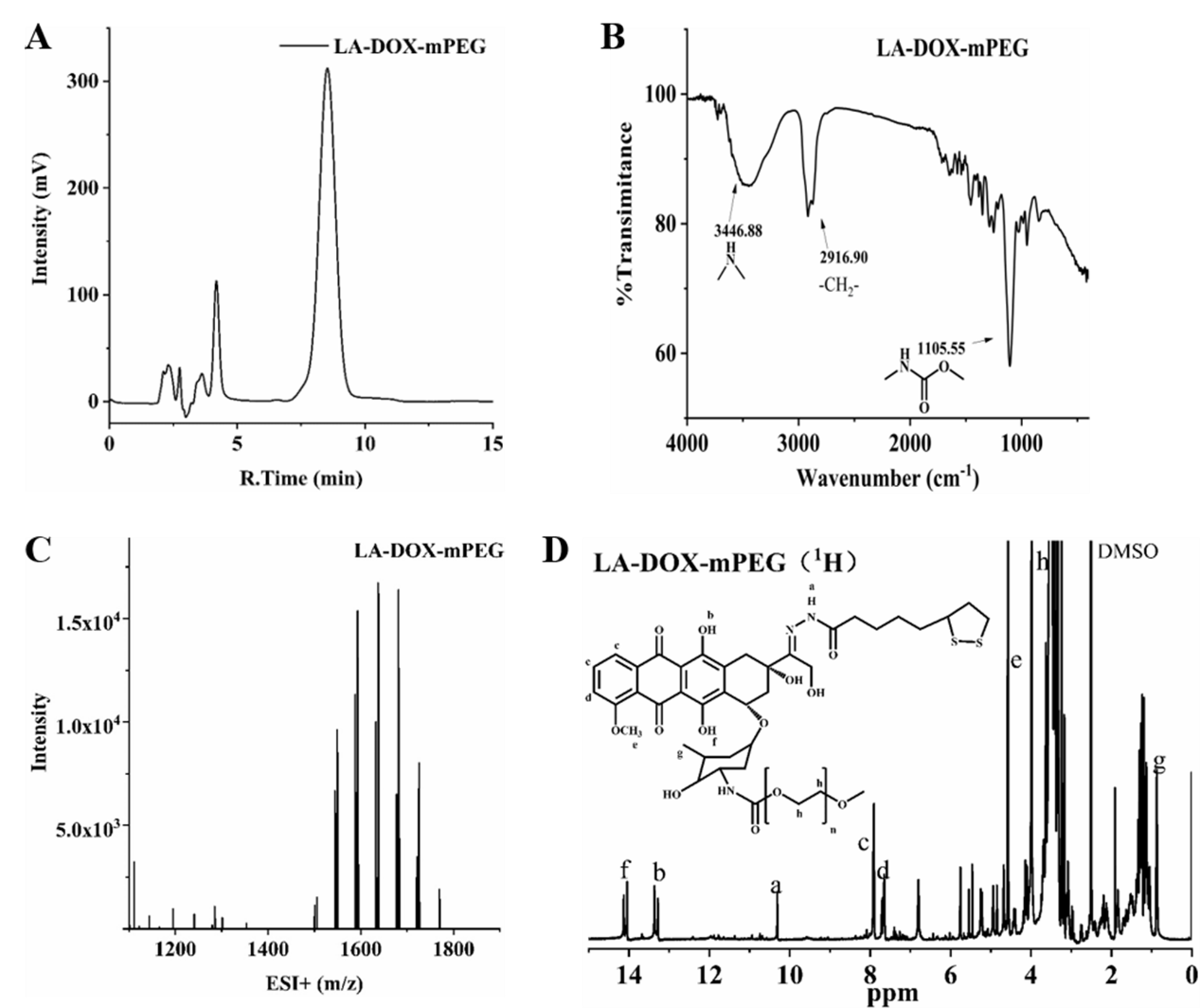


**Figure S5.** Characterization of LA-Dox-mPEG. A) HPLC chromatograms, B) FT-IR, C) MS spectra, D) ^1^H-NMR (DMSO-d_6_) of LA-Dox-mPEG.


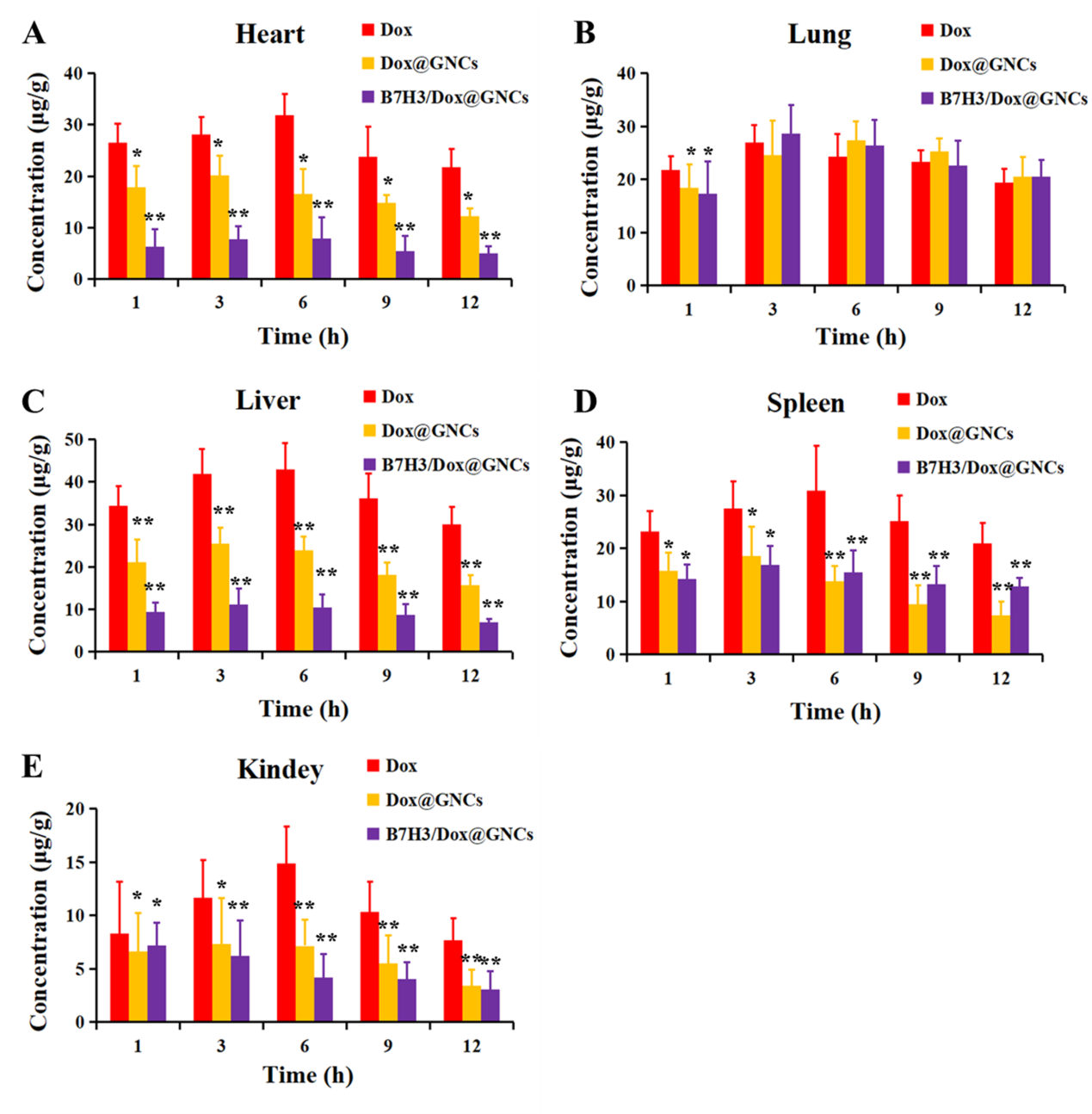


**Figure S6.** *In vivo* biodistribution of Dox, Dox@GNCs, and B7H3/Dox@GNCs. B) Biodistribution profiles of Dox in A) heart, B) Lung, C) Liver, D) Spleen, and E) Kindey of NSCLCs tumor-bearing mice following intravenous injection of Dox·HCl, Dox@GNCs, and B7H3/Dox@GNCs at Dox dose of 3 mg/kg for different time. Data are Mean ± SD (n=3), ^*^*P* ﹤0.05 and ^**^*P* ﹤0.01, (one-sample t-test) versus Dox group.
